# Infrared Multiple Photon Dissociation Spectroscopy of Hydrated Cobalt Anions Doped with Carbon Dioxide CoCO_2_(H_2_O)_*n*_^−^, *n*=1–10, in the C−O Stretch Region

**DOI:** 10.1002/chem.201904182

**Published:** 2020-01-16

**Authors:** Erik Barwa, Milan Ončák, Tobias F. Pascher, Andreas Herburger, Christian van der Linde, Martin K. Beyer

**Affiliations:** ^1^ Institut für Ionenphysik und Angewandte Physik Universität Innsbruck Technikerstraße 25 6020 Innsbruck Austria

**Keywords:** activation, carbon dioxide, catalysis, cobalt, IR spectroscopy

## Abstract

We investigate anionic [Co,CO_2_,*n*H_2_O]^−^ clusters as model systems for the electrochemical activation of CO_2_ by infrared multiple photon dissociation (IRMPD) spectroscopy in the range of 1250–2234 cm^−1^ using an FT‐ICR mass spectrometer. We show that both CO_2_ and H_2_O are activated in a significant fraction of the [Co,CO_2_,H_2_O]^−^ clusters since it dissociates by CO loss, and the IR spectrum exhibits the characteristic C−O stretching frequency. About 25 % of the ion population can be dissociated by pumping the C−O stretching mode. With the help of quantum chemical calculations, we assign the structure of this ion as Co(CO)(OH)_2_^−^. However, calculations find Co(HCOO)(OH)^−^ as the global minimum, which is stable against IRMPD under the conditions of our experiment. Weak features around 1590–1730 cm^−1^ are most likely due to higher lying isomers of the composition Co(HOCO)(OH)^−^. Upon additional hydration, all species [Co,CO_2_,*n*H_2_O]^−^, *n*≥2, undergo IRMPD through loss of H_2_O molecules as a relatively weakly bound messenger. The main spectral features are the C−O stretching mode of the CO ligand around 1900 cm^−1^, the water bending mode mixed with the antisymmetric C−O stretching mode of the HCOO^−^ ligand around 1580–1730 cm^−1^, and the symmetric C−O stretching mode of the HCOO^−^ ligand around 1300 cm^−1^. A weak feature above 2000 cm^−1^ is assigned to water combination bands. The spectral assignment clearly indicates the presence of at least two distinct isomers for *n* ≥2.

## Graphical abstract

**Unexpected structural variety**: IR spectroscopy reveals different motifs of activated carbon dioxide in gas‐phase clusters of composition CoCO_2_(H_2_O)_*n*_^−^, *n*≤10. The dominant isomer class features a formate ligand, but a carbonyl‐dihydroxide structure with the metal inserted into the C−O bond of carbon dioxide is also abundant (see figure).

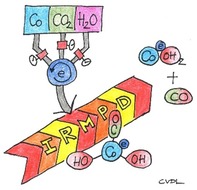

## Introduction

Carbon dioxide as the most important greenhouse gas in the Earth's atmosphere is currently intensely investigated.^1^ The electrochemical route of activation involves the carbon dioxide radical anion CO_2_^−^ as a short‐lived intermediate.^2^, ^3^ It is well known that CO_2_^−^ is metastable and undergoes autodetachment with a measured lifetime of up to milliseconds.^4^, ^5^, ^6^, ^7^ This has been repeatedly confirmed by quantum chemical calculations.^3^, ^7^, ^8^, ^9^ In interaction with a rare gas matrix^10^ or a solvation shell such as (CO_2_)_*n*_^−[6, 11, 12]^ or CO_2_(H_2_O)_*n*_^−^,^13^, ^14^ the radical anion is stabilized.^4^ The same is true in a salt environment where the interaction with positive charge centers is responsible for the stabilization.^15^, ^16^ In the interaction of CO_2_ with metal ions, electron transfer from the metal to the electrophilic carbon atom can occur spontaneously, leading to complexes of the metal center with CO_2_^−^.^4^, ^17^, ^18^, ^19^, ^20^ When a single bond is formed between the metal and the carbon atom, as observed for example, with the nickel group, coinage metal, or bismuth anions, the excess charge in this metalloformate η^1^‐(C) complex, MCO_2_^−^, is delocalized over the whole molecular ion.^21^, ^22^

Organometallic complexes of transition metals like cobalt can play an important role in catalytic reductions of CO_2_,^23^ a key step in carbon capture and usage (CCU) processes. In the gas phase, the reverse reaction, CO oxidation leading to CO_2_, has been observed with anionic cobalt oxide clusters.^24^ Decomposition reactions of copper formate revealed important elementary steps in the transformation of CO_2_ to HCOOH.^25^ Schwarz has recently summarized the mechanistic insight into CO_2_ activation derived from gas‐phase studies, combining experiment and theory.^26^

Vibrational spectroscopy is a powerful method for structural analysis in the gas phase.^4^ Vibrational spectra of Co_*n*_^+^(CH_3_OH)_3_ (*n=*1–3) were measured by IR photodissociation spectroscopy.^27^ Anionic cobalt clusters doped with methanol, ethanol, or propanol molecules were probed by IR spectroscopy in the O−H stretch region.^28^ Cobalt carbonyl cations Co(CO)_*n*_^+^ (*n=*1–9) were investigated in an Ar tagging experiment by the group of Duncan, finding one strong absorption for *n=*1 at 2156 cm^−1^.^29^ Cationic metal–CO_2_ complexes M^+^(CO_2_)_*n*_ (M=Mg, Al, Si, V, Fe, Co, Ni, Rh, Ir) have been extensively investigated in the past decades,^30^, ^31^, ^32^, ^33^, ^34^, ^35^, ^36^, ^37^, ^38^, ^39^, ^40^ and also anionic species M^−^(CO_2_)_*n*_ (M=Ti, Mn, Fe, Co, Ni, Cu, Ag, Au, Sn, Bi) have received considerable attention, foremost by the group of Weber.^41^, ^42^, ^43^, ^44^, ^45^, ^46^, ^47^, ^48^, ^49^, ^50^, ^51^ Generally, the anionic CO_2_^−^ stretching vibrations shift to the red compared to neutral CO_2_ vibrations.^4^, ^52^ CO_2_ as a ligand was also investigated as metal oxides, NbO_2_^+^(CO_2_)_*n*_ and TaO_2_^+^(CO_2_)_*n*_ by Mackenzie and co‐workers.^53^ Photoelectron spectroscopy by the Bowen group revealed CO_2_ activation upon attachment to anionic cobalt pyridine complexes^54^ and provided a different look on anionic coinage metal complexes with CO_2_.^55^

The above‐mentioned IR study of Co(CO_2_)_*n*_^−^ showed that Co forms a core with two negatively charged CO_2_ molecules attached via a bidentate motif, forming a twisted butterfly arrangement. Further CO_2_ molecules surround this core.^43^ A very interesting study on cooperative effects which are operative during metal insertion into the C=O bond of CO_2_ has been performed recently by the group of Weber with Ti^−^(CO_2_)_*n*_.^51^ Insertion of neutral Ti into the C=O bond of CO_2_ had been predicted by quantum chemical calculations.^56^

In an environmentally benign chemical process, water is the ideal solvent. It is, therefore, important to understand cooperative effects during the activation of CO_2_ in the presence of water molecules. We have recently demonstrated C−H,^57^ C−C,^58^, ^59^, ^60^ C−S^61^ bond formation and protonation reactions^62^ with CO_2_^−^(H_2_O)_*n*_ clusters in the gas phase. Nanocalorimetry revealed important details about the thermochemistry of the carbon dioxide radical anion, in particular, its hydration enthalpy.^63^, ^64^ Raman spectroscopy of CO_2_^−^ in bulk aqueous solution^65^ places the symmetric stretching mode of hydrated CO_2_^−^ at 1298 cm^−1^. In our recent IR study on gas phase clusters CO_2_^−^(H_2_O)_*n*_, we observed very similar values already around *n=*20.^14^ Some hydrated metal ions M^+^(H_2_O)_*n*_, M=Mg, Cr, Co, pick up exactly one CO_2_ molecule, indicating that electron transfer from the metal to carbon dioxide takes place.^66^, ^67^, ^68^ In the case of magnesium, the electron is already present in the hydration shell, detached from the metal center, as recently confirmed by electronic spectroscopy of Mg^+^(H_2_O)_*n*_.^69^

For the structural analysis of hydrated metal ions M(H_2_O)_*n*_ (M=Li^+^, Na^+^, Mg^+^, Mg^2+^, Al^+^, Ca^2+^, Co^+^, Co^2+^, Cu^+^, Ag^+^, Cs^+^, Ba^2+^, Tm^3+^, La^3+^), a series of infrared photodissociation studies are available.^70^, ^71^, ^72^, ^73^, ^74^, ^75^, ^76^, ^77^ Pure cationic cobalt clusters Co_*n*_^+^Ar were investigated spectroscopically by argon tagging.^78^ Herein, we report the first IR multiphoton dissociation (IRMPD) study investigating CO_2_ attached to a metal anion solvated with water. The spectra of isolated CoCO_2_(H_2_O)_*n*_^−^, *n=*1–10, clusters along with quantum chemical calculations provide clear evidence of CO_2_ and H_2_O bond rearrangements already for the CoCO_2_H_2_O^−^ ion.

## Experimental and Theoretical Methods

The experiments were performed on a modified 4.7 T FT‐ICR Bruker/Spectrospin CMS47X mass spectrometer^64^, ^79^, ^80^, ^81^, ^82^ equipped with a Bruker infinity cell.^83^ Ions are produced in an external laser vaporization source^84^, ^85^ with a 30 Hz pulsed frequency doubled Nd:YAG laser (Litron Nano S 60‐30). A gas mixture of He, H_2_O, and CO_2_ is expanded through a homebuilt piezoelectric valve. The laser is focused on a rotating Co target, producing a hot plasma, which is cooled by supersonic jet expansion. These ions are guided through a system of electrostatic lenses passing three differential pumping stages to the center of the ICR cell^86^ where they are stored and mass selected in a 4.7 T magnetic field^87^ under ultra‐high vacuum (≈10^−10^ mbar) conditions. A copper shield, which is cooled by liquid nitrogen to *T* ≈80 K, surrounds the cell^88^, ^89^ to minimize the amount of black body infrared radiative dissociation (BIRD).^90^, ^91^, ^92^, ^93^, ^94^, ^95^, ^96^, ^97^, ^98^, ^99^

From the rear side of the magnet, a tunable IR OPO laser system (EKSPLA NT273‐XIR) is coupled into the cell through a CaF_2_ window.^100^ When absorption events lead to photodissociation,^101^ they are detected by the experiment. The measurements were performed in the range of 1250–2234 cm^−1^ where characteristic C−O stretching modes are typically observed. Details on the experimental laser setup can be found elsewhere.^14^, ^100^ The present experiments are lacking information on the number of photons required for dissociation, thus we determine the IRMPD yield, which is total photofragment intensity divided by total ion intensity, irradiation time and laser power. In contrast to the usual definition of IRMPD yield,^102^ we also include the irradiation time, since we adjust it to avoid saturation effects and to increase the signal‐to‐noise ratio of weak bands. As already mentioned above, fragments like CO_2_^.−^ and CO_2_^.−^(H_2_O) cannot be detected,^103^ because the excess electron undergoes autodetachment. However, no signal loss was detected in the present experiment, implying that the decomposition into fragments like CO_2_^.−^ and CO_2_^.−^(H_2_O) does not take place to a significant extent.

Structure and properties of CoCO_2_(H_2_O)_*n*_^−^, *n=*1–10, were studied using methods of theoretical chemistry at the B3LYP/def2TZVPP level of theory. Benchmark calculations with respect to CCSD(T) results for the most stable isomers of *n=*1 can be found in Tables S1 and S2.

The CoCO_2_^−^ ion exhibits either a metalloformate η^1^‐(C) motif or the linear OCoCO^−^ inserted structure. Starting with those, we added a water molecule and constructed several isomers with both, an intact and an activated water molecule, resulting in 14 stable structures for the CoCO_2_H_2_O^−^ ion. By adding successive water molecules to various positions and optimizing the structures, we created structures for clusters with up to four water molecules. For seven selected structures, further solvation with up to a total of 10 water molecules was performed. Vibrational spectra are modeled by using Gaussian broadening with a full width at half maximum (FWHM) of 20 cm^−1^ and scaled by a factor of 0.96. Wavefunction stabilization was performed for every calculation, with internal instability issues found in more than 20 % of calculated structures. All considered structures represent local minima. Transition states are verified through intrinsic reaction coordinate (IRC) calculations. For some transition states, starting points with a small offset along the normal vector of the corresponding imaginary frequency with subsequent steepest decent optimization had to be used to make the IRC calculations work. The Gaussian 16 software was employed for all calculations.^104^

## Results and Discussion

### Bare CoCO_2_^−^

We start our discussion with the non‐hydrated ion, CoCO_2_^−^. In the experiment, no fragments are observed in the investigated wavelength region even after irradiating for 20 s. This is in agreement with the results of Knurr et al.^43^ Dissociation to Co^−^ and CO_2_, the lowest energy fragmentation pathway, requires 73 kJ mol^−1^, calculated at the B3LYP/def2TZVPP level. IRMPD is inefficient in small systems with high binding energy, since the molecule undergoes radiative cooling before dissociation.

### Monohydrated CoCO_2_H_2_O^−^

**Figure 1:**
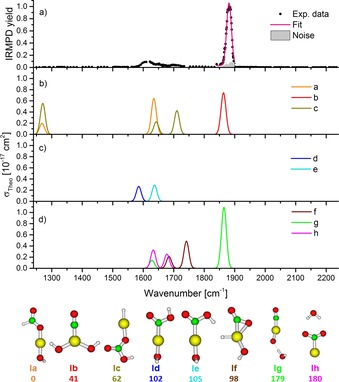
Comparison of a) measured IRMPD spectrum for CoCO_2_H_2_O^−^ resulting in CO loss with b–d) the calculated absorption cross section *σ*_theo_ for isomers **Ia–h**. The main band in the experiment was fitted with a Gaussian to determine the peak position. Geometry optimization and frequency calculation for each isomer was performed at the B3LYP/def2TZVPP level of theory. Relative energy of isomers is given in kJ mol^−1^ including zero‐point correction.

The absorption spectrum of the monohydrated ion, CoCO_2_H_2_O^−^, is shown in Figure 1 a. The only detected fragment is CoOH_2_O^−^ formed in reaction (1), in which *m* is the number of photons:

(1) $$\mathrm{CoCO}{}_{2}H{}_{2}O{}^{-}+mh\nu {}_{\mathrm{IR}}\to \mathrm{CoOH}{}_{2}O{}^{-}+\mathrm{CO}$$

In the measured IRMPD spectrum of CoCO_2_H_2_O^−^, the absorption maximum appears at 1881 cm^−1^. A less intense broad band was observed in the 1570–1730 cm^−1^ region. The absorption saturates upon longer irradiation at the maximum, but only 25 % of the precursor ions dissociate. Laser misalignment can be ruled out, since other ions could be almost fully depleted with the same laser alignment. This indicates that additional isomers are present with an abundance of ≈75 %, which do not absorb at this wavelength.

Quantum chemical calculations of CoCO_2_H_2_O^−^ reveal a rich structural diversity. The most stable structure is isomer **Ia**, with Co(OH)(HCO_2_)^−^ structure, in which both H_2_O and CO_2_ are activated, see Figure 1. Isomer **Ib** with cobalt inserted in the C=O bond is less stable by 41 kJ mol^−1^. Further isomers with activated H_2_O, HCo(HCO_3_)^−^ (**Ic**), HOCo(HOCO)^−^ (**Id**, **Ie**), and HCoOH(CO_2_)^−^ (**If**) lie even higher in energy. Two isomers with intact H_2_O (**Ig**) and both intact H_2_O and CO_2_ (**Ih**) lie about 180 kJ mol^−1^ above **Ia**.

**Figure 2:**
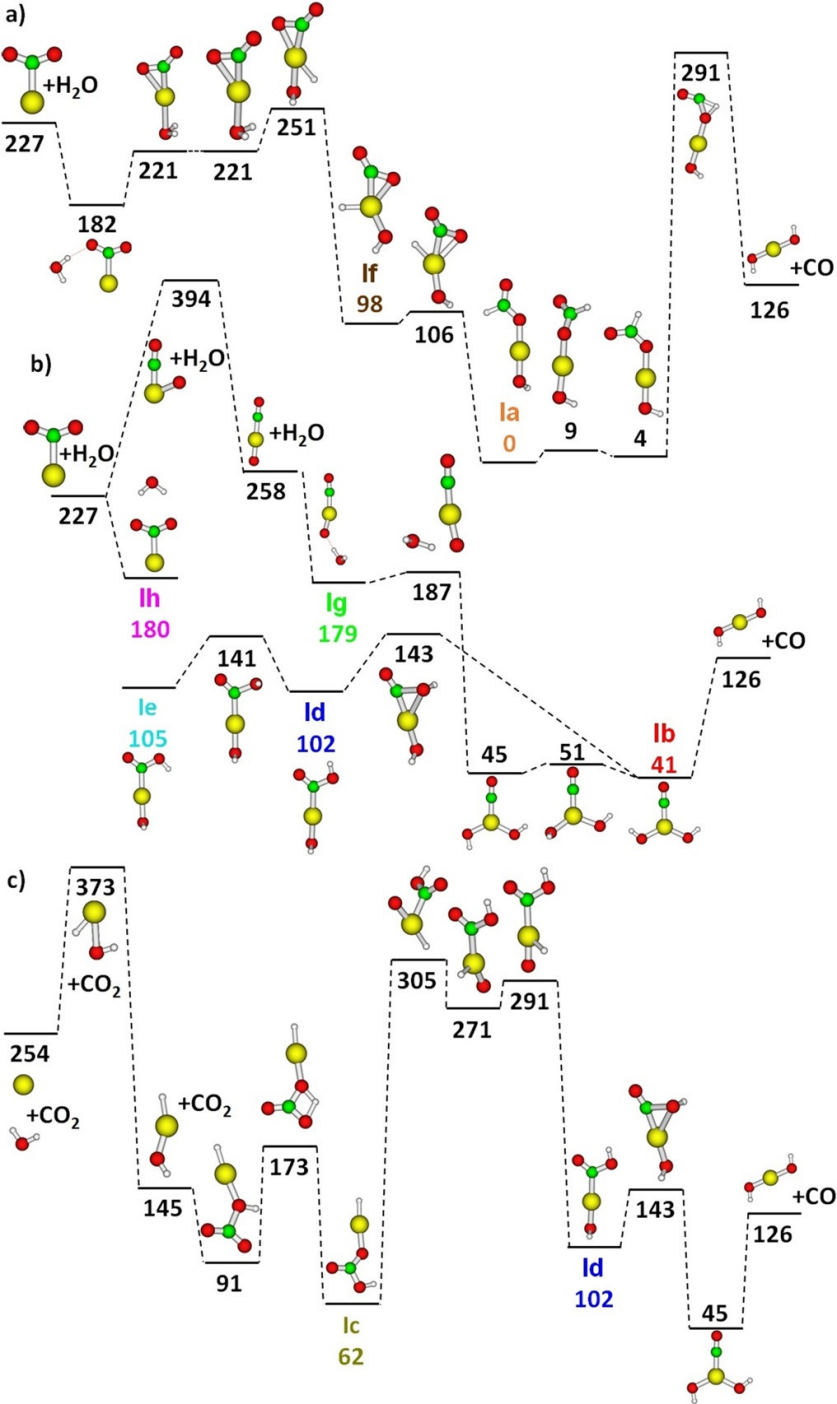
Potential energy surface for CoCO_2_H_2_O^−^, with relative energy including zero‐point correction in kJ mol^−1^. Calculated at the B3LYP/def2TZVPP level of theory.

Figure 2 shows the potential energy surface of possible CO loss reactions for the CoCO_2_H_2_O^−^ ion. Figure 2 a reveals a low water activation energy on CoCO_2_^−^ of 24 kJ mol^−1^ relative to the entrance channel, transferring a hydrogen atom metal‐mediated to CO_2_ and eventually creating the most stable Co(OH)(HCO_2_)^−^ structure (**Ia**), with **If** as an intermediate. Another possible pathway can be seen in Figure 2 b, in which CO_2_ activation in the absence of water requires 167 kJ mol^−1^ relative to the entrance channel. As soon as water is added, the OCCoO(H_2_O)^−^ structure (**Ig**) is formed. From there, water activation proceeds readily over a small barrier, and the path opens to form the OCCo(OH)_2_^−^ ion (**Ib**). Water activation on bare Co^−^ requires 119 kJ mol^−1^ (Figure 2 c). CO_2_ can then be further activated over a barrier of about 80 kJ mol^−1^ forming isomer **Ic**. A potential energy barrier of 243 kJ mol^−1^ needs to be overcome for isomerization to **Id** with Co(OH)(HCO_2_)_2_^−^ structure.

The most prominent spectral feature in the experiment at 1881 cm^−1^ can be reproduced by the C=O vibration in both **Ib** and **Ig** isomers (Figure 1). However, isomerization of **Ig** to **Ib** faces a barrier of only 8 kJ mol^−1^ (Figure 2 b) and is thus not expected to survive in the ICR cell. In the **Ib** structure, a CO group is present and will readily dissociate after absorption of 3–4 photons at 1881 cm^−1^. We thus assign the 1881 cm^−1^ band exclusively to isomer **Ib**. The experiment indicates that this isomer forms about 25 % of the total ion abundance, estimated from the IRMPD yield in saturation.

The weaker absorption band observed experimentally at 1570–1730 cm^−1^ lies in the range of the H_2_O bending mode and the antisymmetric stretching mode of CO_2_^−^.^14^ The presence of an intact water molecule, isomers **Ig** and **Ih**, can be ruled out. According to Figure 2 these ions are expected to dissociate by loss of water, which is not observed in the experiment. The remaining calculated isomers **Ia**, **Ic–f** all exhibit vibrational modes in this region. The presence of the most stable isomer **Ia** is probable, also due to its vibration at ≈1300 cm^−1^ observed for *n*>1 (see below). The CO loss energy is calculated to be 126 kJ mol^−1^ with respect to isomer **Ia**, but it requires a rearrangement with a barrier of 291 kJ mol^−1^. For that reason, it is not plausible that **Ia** contributes to the observed photodissociation spectrum, as approximately 15 photons would be required. Similarly, isomer **Ic** is topologically well separated from the CO loss pathway and CO_2_ loss would be the most probable channel here.

Only isomers **Id** and **Ie** can thus account for the broad weak feature. These isomers feature a HOCO ligand, with absorptions in the relevant spectral region. Both face a barrier around 40 kJ mol^−1^ against rearrangement to isomer **Ib**, and the barriers lie above the CO loss channel, Figure 2 b. Isomerization to **Ib** will, therefore, be immediately followed by CO loss. The barrier corresponds to the absorption of 2–3 photons. Depending on the orientation of the ligand in **Id** and **Ie** and dynamic effects, the spectrum may exhibit the observed broad structure, given the high conformational flexibility of the HOCO ligand. Since relatively few photons are required for dissociation of **Id** and **Ie**, a low abundance of these isomers is sufficient to cause the observed features.

We therefore conclude that from the calculated isomers, only **Ib**, **Id** and **Ie** contribute to the observed spectrum. Isomer **Ia** is very likely present, even as the most abundant isomer, but it does not lead to an IRMPD signal under the conditions of our experiment.

### Dihydrated CoCO_2_(H_2_O)_2_^−^

For clusters with two water molecules, water evaporation is exclusively observed, reaction (2) with *n=*2. The most intense absorption band shifts to the blue, and additional bands arise at both ends of the spectrum.

(2) $$\mathrm{CoCO}{}_{2}\left(H{}_{2}O\right){}_{n}{}^{-}+mh\nu {}_{\mathrm{IR}}\to \mathrm{CoCO}{}_{2}\left(H{}_{2}O\right){}_{n-1}{}^{-}+H{}_{2}O$$

**Figure 3:**
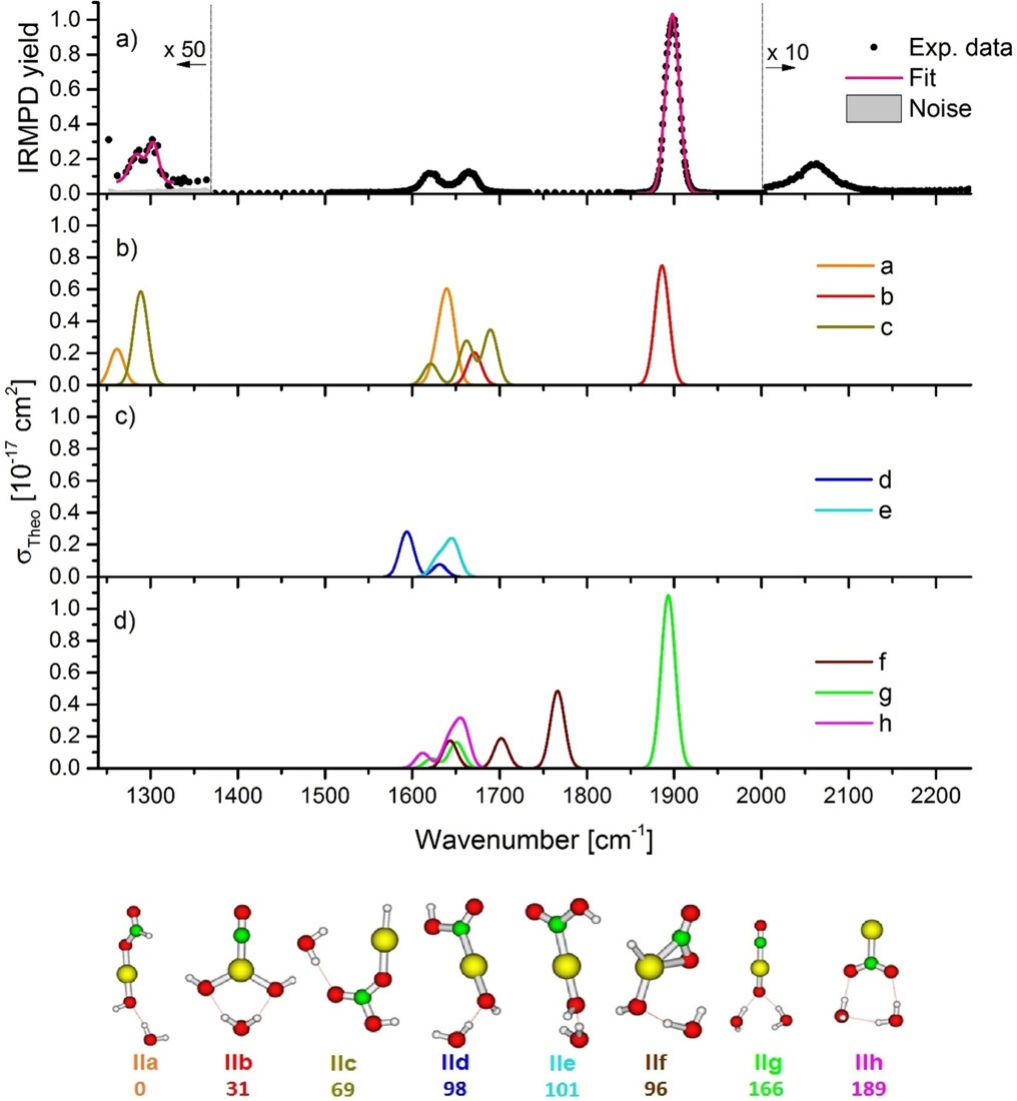
Comparison of a) measured IRMPD spectrum for CoCO_2_(H_2_O)_2_^−^ with H_2_O loss with b–d) calculated absorption cross section *σ*_theo_ for isomers **IIa–h**. The main band in the experiment was fitted with a Gaussian. Geometry optimization and frequency calculation for each isomer was performed at the B3LYP/def2TZVPP level of theory. Relative energy of isomers is given in kJ mol^−1^ including zero‐point correction.

The features from the monohydrated species are again observed, Figure 3. The absorption maximum in the IRMPD spectrum lies at 1898 cm^−1^, shifted by about 18 cm^−1^ to the blue, and roughly an order of magnitude more intense compared to the *n=*1 spectrum. The higher intensity is due to the fact that H_2_O loss requires less energy than the loss of a CO molecule, that is, only about two photons. At longer irradiation times, CoOH_2_O^−^ is formed by secondary fragmentation of CoCO_2_H_2_O^−^. To avoid saturation effects and secondary fragmentation, this strong band around 1900 cm^−1^ is measured with shorter irradiation time than the rest of the spectrum.

In the region of 1500–1700 cm^−1^, two clearly visible bands at ≈1622 and ≈1665 cm^−1^ are observed for *n=*2. Further, two new absorption bands are observed, a very weak transition between 1272 and 1314 cm^−1^ and a band around 2060 cm^−1^. The isomer absorbing in the former region seems to be present only in very little amount in our experiment. Even after irradiation times as long as 10 s, only ≈2 % of the ions dissociate due to laser irradiation. This band might arise due to the symmetric C−O stretching mode of an HCO_2_^−^ ligand, which lies at 1314 cm^−1^ in HCOO^−^(Ar).^105^, ^106^ In a recent study by Weber on [Ti(CO_2_)_*n*_]^−^, in which titanium inserts into a C=O bond, a small band observed at 2056 cm^−1^ was assigned to oxalato ligands, which can be ruled out here.^51^

DFT calculations predict very similar structures compared to the case of one water molecule. The most stable isomer **IIa** has a (H_2_O)(OH)Co(HCO_2_)^−^ structure, that is, CO_2_ and one H_2_O are activated. Isomer **IIb** with an inserted metal in the C=O bond and an activated H_2_O is less stable by only 31 kJ mol^−1^. Further isomers lie at least ≈70 kJ mol^−1^ higher in energy.

**Figure 4:**
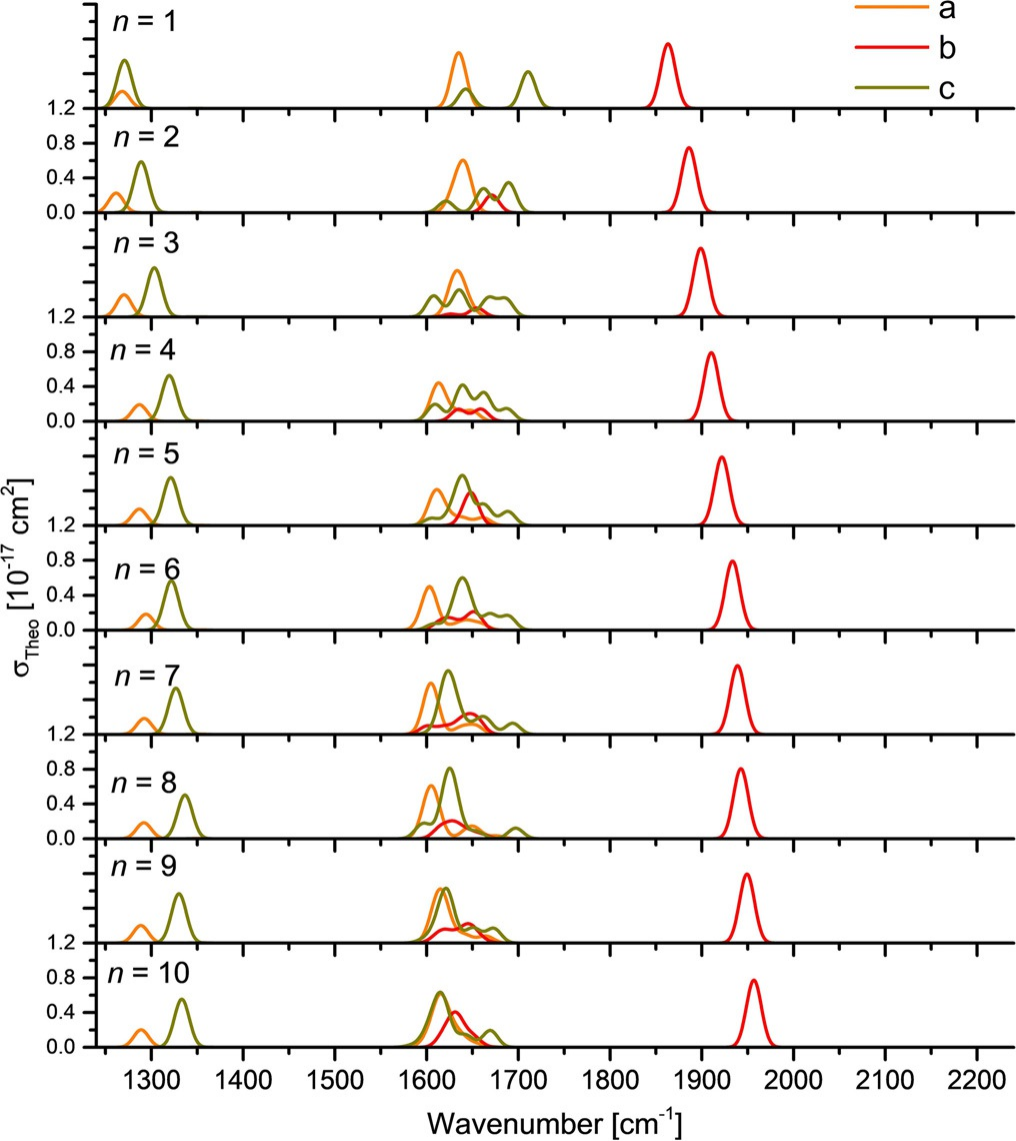
IR spectra of CoCO_2_(H_2_O)_*n*_^−^, 1≤*n*≤10, isomers **a‐**‐**c** (see Figures 1, Figure 2, and Figure S4, Supporting Information, for the respective structures) calculated at the B3LYP/def2TZVPP level of theory. Spectra of less stable isomers are shown in Figure S1, Supporting Information.

As seen in Figures 3, 4, and Figure S1, Supporting Information, calculated IR spectra do not change much when passing from one to two water molecules. The most intense band in the experiment at 1898 cm^−1^ results from the C=O vibration in isomer **IIb**. The absorption at ≈1580–1700 cm^−1^ is due to a mixture of the bending mode of the intact H_2_O molecule and the antisymmetric C−O stretching mode in the HCO_2_ ligand, with contributions from various isomers, for example, the C−O stretch in **IIc‐e** as well as the water bend in **IIa** or **IIb**. In isomers **IIc** and **IIf**, the frequencies corresponding to the Co−H vibration lie between 1650 and 1800 cm^−1^.

The small absorption at low energies can be assigned to either isomer **IIa** or **IIc**. Depending on the angle of the (HCO_2_) complex in **IIa**, the absorption might shift even more to higher energies as seen in Figure S2, Supporting Information. The presence of an exotic Co(OH)(H_2_O)**⋅⋅⋅**HCO_2_^−^ complex with a relative energy of 24 kJ mol^−1^ could also account for the observed band, see Figure S2, Supporting Information. However, formation of such an isomer does not correspond to the observed water loss within the IRMPD process.

With respect to the experimentally measured band at 2060 cm^−1^, no calculated isomer features harmonic vibrational modes near this wavenumber. Our excited states calculations at the equation of motion‐coupled cluster singles and doubles (EOM‐CCSD) level show that there are also no low‐lying electronically excited states in the IR region. Such states are calculated in the OCoCO^−^ ion but disappear upon water activation. Most likely, the band origins from overtones and combination bands of lower‐lying transitions.

### Larger hydrated species

Clusters with *n*>2 also evaporate a single water molecule upon resonant IR irradiation, reaction (2). Saturation effects become more evident with increasing cluster size, and the effect on the band shape of the absorption at 1900 cm^−1^ is shown for *n=*3 with two different irradiation times *t*_IR_ in Figure S3, Supporting Information.

**Figure 5:**
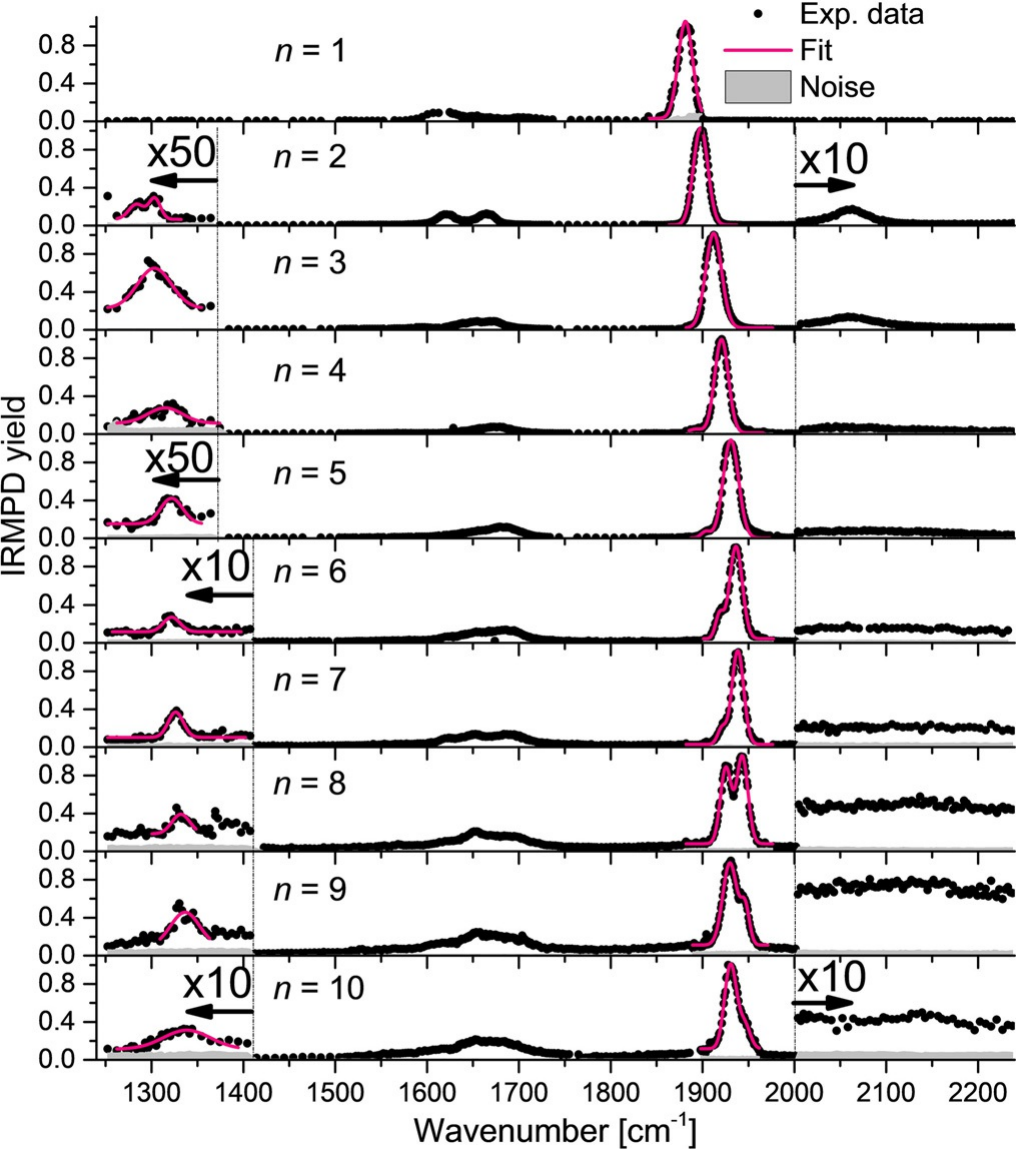
Infrared multiple photon dissociation spectra of CoCO_2_(H_2_O)_*n*_^−^ for 1≤*n*≤10 in the 1250–2234 cm^−1^ region. An irradiation time of 20 s is used for *n=*1. For *n*>1 ions are generally irradiated for 3 s, with exception of the main peak, which is measured with 1 s for *n=*2 and 0.5 s for *n*>2 to avoid saturation.

**Figure 6:**
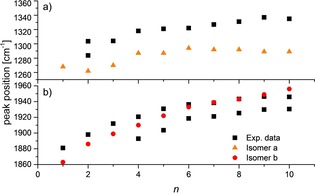
The peak positions of the Gauss fits of each absorption band compared with the theoretical value for cluster sizes *n=*1–10 in the following regions: a) 1303–1337 cm^−1^, b) 1881–1938 cm^−1^. Starting from *n=*4, two Gaussians are required for the fit in b), see Figure 5. See Figure S5, Supporting Information, for further details of the evolution of the region 1881–1938 cm^−1^.

The spectra for *n=*1–10 are shown in Figure 5, with the spectra for *n=*1, 2 included for comparison. Generally, one can see a blueshift of the bands at ≈1300 and ≈1900 cm^−1^, whereas the other two bands do not exhibit a systematic shift. These shifts are compared in Figure 6, in which the evolution of absorption maxima with respect to the cluster size is shown. For the band at ≈1300 cm^−1^, experimental data shows an average shift of ≈4 cm^−1^ per water molecule. This shift is reproduced in the calculations by the vibrations of isomers **Ia‐Xa** with an average shift of ≈3 cm^−1^ per water molecule (Figure 6 a). The corresponding structures are shown in Figure S4, Supporting Information.

The most intense absorption is found for all cluster sizes between 1860 and 1960 cm^−1^ and shifts to the blue with increasing *n*. As mentioned above, this band corresponds to the C=O vibration in isomer **b**, and its shift can be well reproduced by our calculations, see Figure 6 b. For *n*≤6, a nearly linear blue shift is observed. As seen in Figure S5, Supporting Information, a shoulder arises on the low‐energy side for *n=*4, which becomes more and more dominant for higher *n*, and two data points are included for these cluster sizes in Figure 6 b. This band is also seen in CO adsorption experiments on a Co surface.^107^ It is also seen as a very weak feature for inserted isomers in Co(CO_2_)_*n*_^−^ by the group of Weber.^43^ We interpret it here as the emergence of a new isomer, most likely involving a hydrated CO group.

The wavenumber region of 1550–1750 cm^−1^ is composed mainly of water vibrations, with minor contributions from the antisymmetric stretching mode of formate. No clear trends can be identified due to several isomers contributing to the spectral envelope. Theoretical calculations do not show any clear trend with respect to the cluster size for any isomer, Figure 4.

The last feature at ≈2060 cm^−1^ exhibits a pronounced band only for 2≤*n*≤5. It does not shift to the blue with increasing *n*. However, the band broadens with increasing *n* so that the band is smeared out for *n*≥6, resulting in a raised baseline as seen in Figure 5. For larger clusters, the band might be explained by a combination of H_2_O bending ν_2_, H_2_O libration ν_L2_, and bending of H_2_O triplets ν_T2_,^108^ as seen before in the spectra of CO_2_^−^(H_2_O)_*n*_.^14^

## Conclusions

We measured IR multiple photon dissociation spectra of the CoCO_2_(H_2_O)_*n*_^−^ systems. As reported before,^43^ the non‐hydrated species CoCO_2_^−^ does not show an IRMPD signal in the wavelength region investigated. Already for *n=*1, the most prominent absorption is characteristic of a metal‐coordinated CO group, which shows that the Co atom has inserted into the C=O bond of CO_2_. However, the spectra also show that multiple isomers are present, and those without a metal coordinated CO seem to prevail. Two isomers featuring a HOCO ligand are most likely responsible for the weak, broad transition around 1570–1730 cm^−1^, since they have absorptions in that region and simple rearrangements allow for the release of CO.

For *n*≥2, all primary IRMPD signals are due to loss of one H_2_O molecule. The probably most abundant isomer class that features a formate ligand is directly evidenced by a band the position of which shifts from 1303 to 1337 cm^−1^ upon hydration with up to 10 H_2_O molecules. The region, which could be indicative of a HOCO ligand, however, is now smeared out by overlapping absorptions due to the water bending and antisymmetric HOCO^−^ or HCOO^−^ stretching modes. The most intense absorption of the C−O stretching mode in the metal inserted isomer shifts to the blue with increasing *n*, from 1881 to 1938 cm^−1^. A weak feature at roughly 2060 cm^−1^, which is assigned to a combination band of low‐lying water modes, smears out with increasing solvation, leading to an elevated baseline for large clusters in this region.

We rationalize the presence of different isomers by the pronounced non‐equilibrium conditions in the ion source. Due to the specific nature of the potential energy surface of the and thus persist under the experimental conditions.

## Conflict of interest

The authors declare no conflict of interest.

## Supporting information

As a service to our authors and readers, this journal provides supporting information supplied by the authors. Such materials are peer reviewed and may be re‐organized for online delivery, but are not copy‐edited or typeset. Technical support issues arising from supporting information (other than missing files) should be addressed to the authors.

Supplementary
