# Supplementary material for: Infrared Multiple Photon Dissociation Spectroscopy of Hydrated Cobalt Anions Doped with Carbon Dioxide CoCO2(H2O)n −, n=1–10, in the C−O Stretch Region
Source: Chemistry. 2020 Jan 16;26(5):1074–81. doi: 10.1002/chem.201904182 (PMC7051846; doi:10.1002/chem.201904182)
Supplement: Supplementary file 1 — Supplementary [file CHEM-26-1074-s001.pdf]

CHEMISTRY
A European Journal

Supporting Information

Infrared Multiple Photon Dissociation Spectroscopy of Hydrated Cobalt Anions Doped with Carbon Dioxide $CoCO_2(H_2O)_n^-$, $n=1–10$, in the C–O Stretch Region

Erik Barwa, Milan Ončák,* Tobias F. Pascher, Andreas Herburger, Christian van der Linde, and Martin K. Beyer*[a]

chem_201904182_sm_miscellaneous_information.pdf

Benchmarking of Theoretical Methods

Table S1: Benchmarking of two different basis sets using the B3LYP method compared with other *ab initio* methods. Single-point calculations in the structure optimized at the B3LYP/def2TZVPP level. Energies are given in kJ/mol.

	B3LYP/def2TZVPP	*B3LYP/aug-cc-pVDZ*	*M06L/def2TZVPP*	*M06/def2TZVPP*	*BMK/def2TZVPP*	*CCSD(T)/def2TZVPP*
Ia	0	0	0	0	0	0
Ib	47	35	14	56	79	26
Ic	65	72	61	56	61	65
Id	102	96	109	107	107	98
Ie	105	97	107	108	111	101
If	111	109	70	121	126	107
Ig	183	171	141	213	245	181
Ih	179	185	173	174	198	240
CO_2 dissociation	162	165	136	149	173	174
CO dissociation	140	132	155	145	137	120

Table S2: Absolute difference between the CCSD(T)/def2TZVPP results and results provided by other methods as well as the other basis set introduced in Table SI1. Energies are given in kJ/mol.

	B3LYP/def2TZVPP	*B3LYP/aug-cc-pVDZ*	*M06L/def2TZVPP*	*M06/def2TZVPP*	*BMK/def2TZVPP*
Ia	0	0	0	0	0
Ib	21	9	12	30	53
Ic	0	7	4	9	4
Id	4	2	10	9	9
Ie	4	3	7	7	10
If	4	1	37	14	19
Ig	2	10	41	32	63
Ih	61	55	67	66	41
CO_2 dissociation	11	8	38	25	0
CO dissociation	20	12	35	24	17
Arithmetic mean	13	11	25	22	22

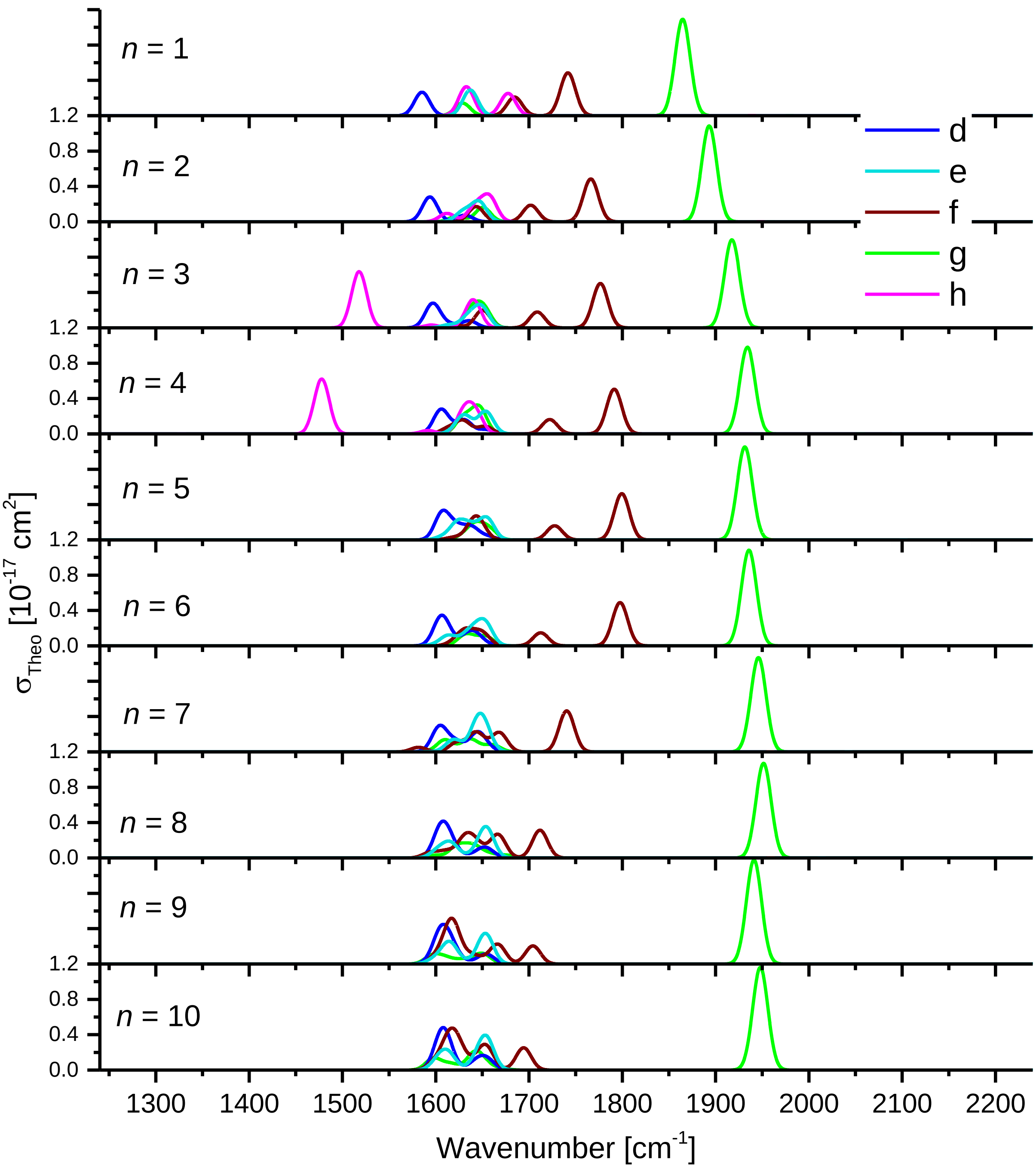

Figure S1: Calculated spectra of $CoCO_2(H_2O)_n^-$ for $1 \leq n \leq 10$ to the coresponding isomers d-h seen in Figure S4. Calculated at the B3LYP/def2TZVPP level of theory.

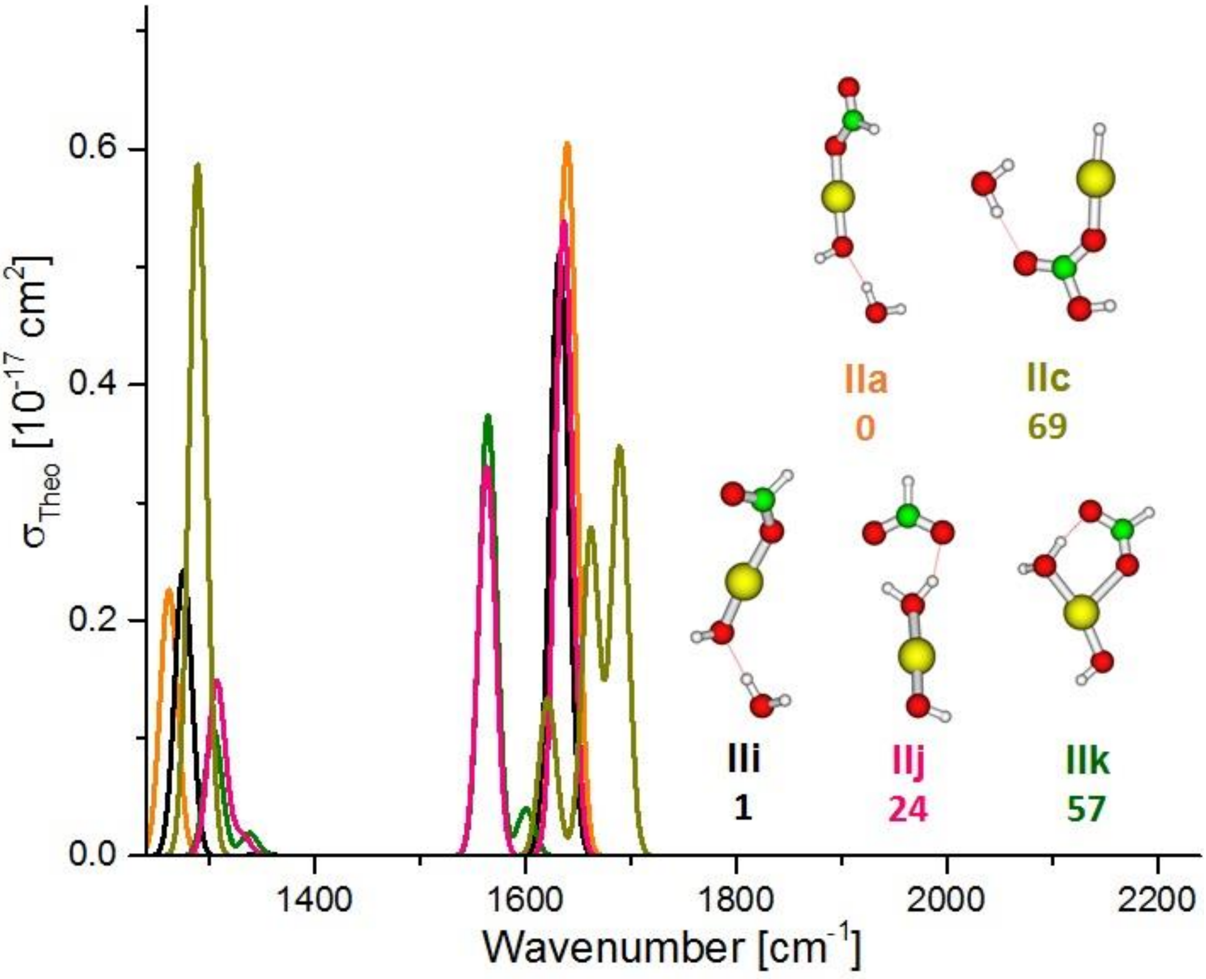

Figure S2: Isomers of $CoCO_2(H_2O)_2^-$ with a low energy absorption band (~1300 cm^{-1}). Calculated on the B3LYP/def2TZVPP level of theory. Relative energy of isomers is given in kJ/mol.

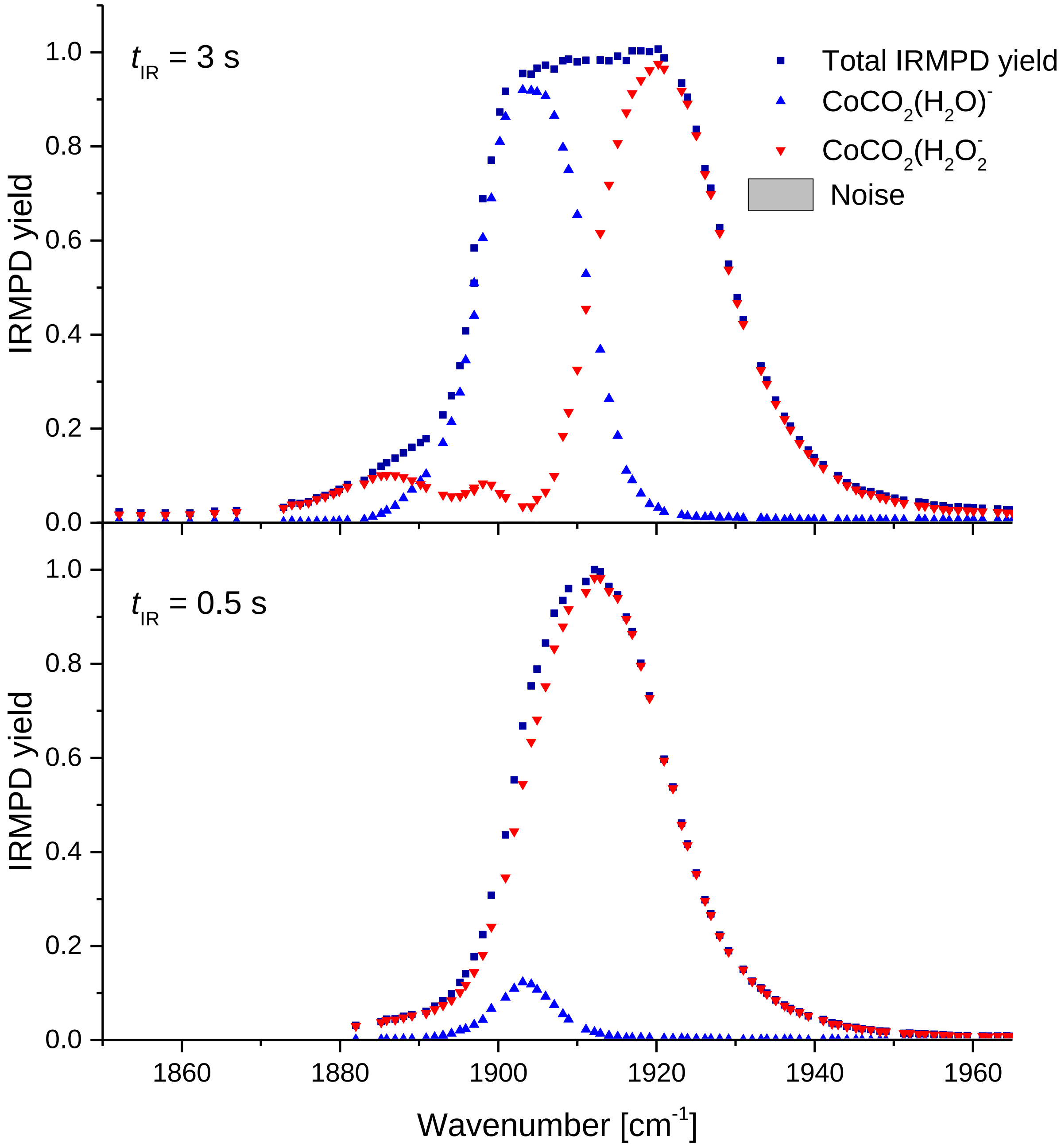

Figure S3: Shape comparison of the main absorption band of $CoCO_2(H_2O)_3^-$ for irradiation times of 3 s and 0.5 s.

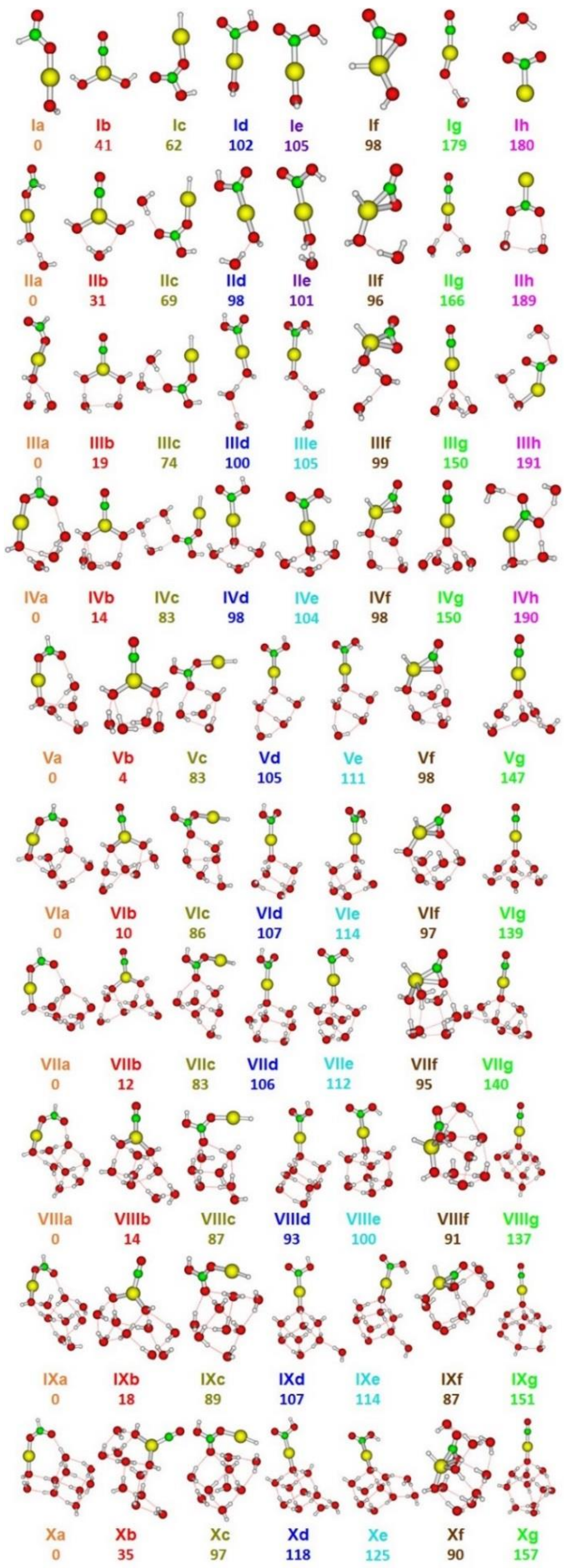

Figure S4: Calculated structures of $CoCO_2(H_2O)_n^-$ for $1 \leq n \leq 10$ with energies given in kJ/mol at the B3LYP/def2TZVPP level of theory.

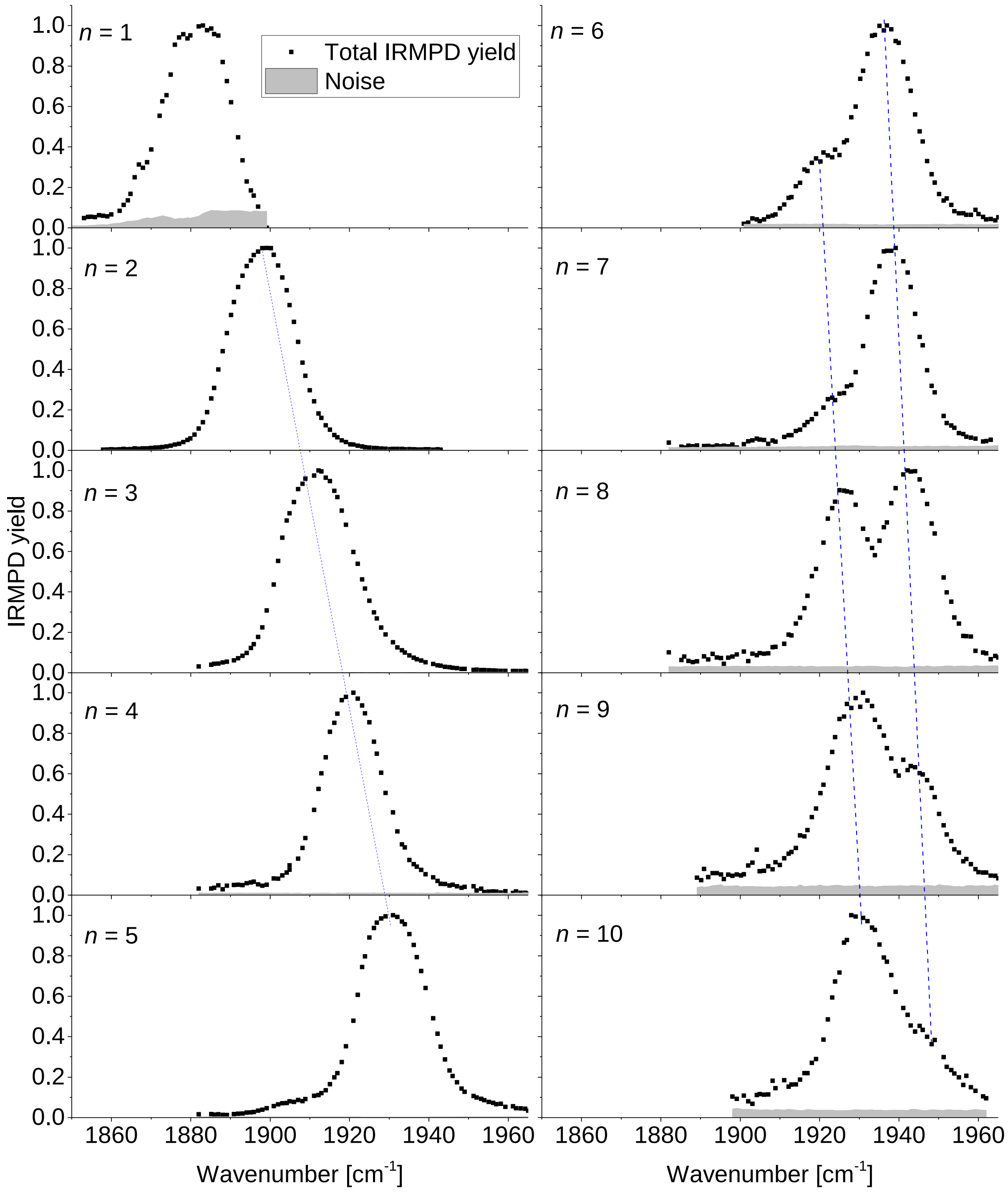

Figure S5: Measured absorption spectra of $CoCO_2(H_2O)_n^-$ in the range of 1850–1970 cm^{-1}. Due to saturation effects, shorter irradiation time of 1 s for n = 2, and 0.5 s for $n \geq 3$ are used for the measurement of this band. The shift to higher energy with increasing n is indicated by the vertical dashed blue line. For $n \geq 6$, a double peak is observed. For $n > 8$, the center of the band is described by the low-energy part of the double peak what can be seen in Figure 6b.

Cartesian coordinates (in Å) and electronic energies (in Hartree) including zero-point energy as optimized at the B3LYP/def2TZVPP level of theory

Ia
-1648.015131
H -1.787931 1.436416 0.097193
O -3.195066 -0.000072 -0.000573
Co 0.859059 -0.085407 -0.001330
C -2.022861 0.350908 0.024329
O -0.992560 -0.417329 -0.024913
O 2.641990 0.280666 -0.082087
H 3.095597 -0.141988 0.653329

Ib
-1647.999527
C 1.432385 -0.000024 -0.000069
Co -0.334135 0.000001 -0.000065
O -1.197734 1.680489 0.000055
O 2.600378 -0.000034 0.000132
O -1.197815 -1.680443 0.000063
H -0.605703 -2.432723 0.000096
H -0.605581 2.432737 0.000075

Ic
-1647.991579
Co -1.381118 -0.763818 -0.000000
H -2.583558 -1.768383 -0.000000
O -0.000000 0.595921 0.000000
C 1.282708 0.563669 0.000000
O 2.053156 -0.375401 0.000000
O 1.834880 1.850666 0.000000
H 1.073214 2.439967 0.000000

Id
-1647.976361
C -1.280113 -0.125577 -0.004119
O -1.966536 1.100363 0.010337
O -1.989992 -1.121143 0.019994
Co 0.702422 -0.014748 -0.019331
O 2.545663 0.049134 -0.070355
H 2.908536 0.079982 0.819979
H -2.906339 0.844837 0.046870

Ie
-1647.975312
C -1.305520 -0.144321 0.005409
O -2.027451 -1.117273 0.003008
Co 0.677225 -0.051007 -0.000969
O 2.517208 0.069383 -0.102845
O -2.000125 1.099770 0.002630
H 2.933688 0.061865 0.761701
H -1.302698 1.766216 0.009657

If
-1647.977763
o -2.297942 0.266050 -0.141087
h -0.214433 -1.816387 0.124121
co -0.514765 -0.300763 0.015064
h -2.741388 0.483680 0.681484
c 1.388315 0.077531 -0.006407
o 2.515905 -0.360621 -0.029808
o 0.847609 1.218086 0.024160

Ig
-1647.946905
C -2.063140 0.158596 -0.005363
Co -0.308069 -0.080183 -0.053289
O 1.293046 -0.592406 0.117356
O -3.224096 0.264936 0.069102
O 3.699884 0.390100 -0.092619
H 2.769801 -0.021195 -0.079738
H 3.776226 0.733507 0.800009

Ih
-1647.946643
O -0.740050 1.139489 0.021527
C -0.278255 -0.000609 0.026918
Co 1.738572 0.000314 -0.010174
O -0.739527 -1.140817 0.021235
O -3.469407 0.000704 -0.024918
H -2.839193 0.737039 -0.016218
H -2.840852 -0.736853 -0.013352

Other structures calculated for the PES
Local Minimum (LM)
Transition State (TS)

Co^-: -1382.797522
Co 0.000000 0.000000 0.000000

CoH_2O^-: -1459.259964
O 2.559581 -0.071817 -0.000024
Co -0.908645 0.002534 -0.000002
H 1.626267 -0.376251 0.000181
H 2.430509 0.882372 0.000062

$HCoOH^-$: -1459.301577
Co -0.001631 -0.429421 -0.000000
H -0.660049 -1.872697 -0.000000
O -0.001631 1.426165 0.000000
H 0.717131 2.057754 0.000000

$Co(OH)_2^-$: -1534.609821
Co -0.000000 0.000258 0.000000
O 1.740325 -0.594637 -0.000000
H 2.320497 0.173505 -0.000000
O -1.740355 0.593924 0.000000
H -2.320255 -0.174773 0.000000

CO: -113.357482
C 0.000000 0.000000 -0.642856
O 0.000000 0.000000 0.482142

CO_2: -188.658741
C -0.000000 -0.000000 -0.045947
O 0.000000 -1.163297 0.017230
O -0.000000 1.163297 0.017230

H_2O: -76.445360
O -0.000000 0.000000 0.117086
H -0.000000 0.761828 -0.468342
H -0.000000 -0.761828 -0.468342

LM $CoCO_2$ (226.6 kJ/mol)
-1571.48348

co -1.056573 0.000026 0.000364
c 0.986377 -0.000005 -0.009475
o 1.412984 -1.145068 0.002939
o 1.413168 1.144986 0.002939

LM OCoCO (258.0 kJ/mol)
-1571.471519
C 0.000000 0.000000 -1.344469
Co 0.000000 0.000000 0.424419
O 0.000000 0.000000 2.096097
O 0.000000 0.000000 -2.520158

TS OCoCO (393.5 kJ/mol)
-1571.419877
O -0.124058 1.345346 -0.000023
C 0.947386 -0.265109 -0.000003
Co -0.799404 -0.282578 0.000012
O 2.111508 -0.192811 -0.000014

LM (90.9 kJ/mol)
-1647.980527
Co -1.585999 0.005665 0.000094
H -3.113721 0.336002 0.000661
O 0.278790 -0.598908 -0.000679
H 0.496201 -1.533931 -0.000358
C 1.692468 0.133397 -0.000001
O 2.579095 -0.704901 0.000459
O 1.552700 1.334382 -0.000136

LM (4.0 kJ/mol)
-1648.013617
C -2.153924 -0.206313 0.000053
O -2.411809 0.989418 -0.000041
O -1.015114 -0.798421 0.000225
Co 0.759792 -0.123081 -0.000076
O 2.466575 0.522351 0.000206
H -2.998412 -0.935882 -0.000577
H 3.090361 -0.209837 -0.000812

LM (46.6 kJ/mol)
-1647.998150
C -0.096983 -1.457031 0.000000
O -0.144390 -2.622364 0.000000
Co 0.000000 0.310897 0.000000
O -1.645727 1.267890 0.000000
O 1.736168 1.067316 0.000000
H -1.443655 2.208147 0.000000

TS (141.4 kJ/mol)
-1647.961275
o 2.522516 0.058612 -0.047688
co 0.680880 -0.046238 -0.029699
c -1.276282 -0.193756 -0.020247
o -2.049661 -1.113192 0.053303
o -1.917986 1.152934 -0.096907
h 2.875499 0.190144 0.837013
h -2.040524 1.433991 0.816675

TS (324.5 kJ/mol)
-1647.891531
o -0.356364 -0.830955 -0.000052
c 0.172214 1.009680 0.000013
co 1.347934 -0.302037 -0.000026
o -0.652209 1.834726 0.000038
o -3.030606 -0.673872 0.000079
h -2.044050 -0.828066 0.000029
h -3.070021 0.285795 0.000095

TS (173.3 kJ/mol)
-1647.949119
o -2.352829 -0.881154 0.000259
h -1.146836 -1.350022 -0.000143
c -1.556108 0.158557 -0.000034
o -1.760503 1.347035 -0.000010
o -0.307356 -0.475083 -0.000482
co 1.582356 0.007828 0.000093
h 3.125377 0.260955 -0.000285

TS (186.7 kJ/mol)
-1647.944009
O -1.895840 1.368276 -0.096949
H -1.998895 0.273358 0.031825
Co -0.027484 -0.327495 -0.067723
O -1.592011 -1.043978 0.143165
C 1.672818 0.156311 -0.013929
O 2.807429 0.416350 0.088641
H -1.852575 1.705958 0.801427

TS CoH2O-
-1459.214868
h 0.510241 1.063182 0.074286
co -0.547225 -0.020228 0.002048
o 1.558439 -0.045998 -0.117887
h 1.797316 -0.149037 0.813501

TS (250.9 kJ/mol)
-1647.919552
o -2.482136 -0.140886 -0.030136
h -1.640311 -1.094698 -0.345751
co -0.497259 0.018123 -0.026887
h -2.746943 -0.373047 0.870212
c 1.421431 -0.121247 0.011078
o 2.434666 -0.791099 0.032106
o 1.208053 1.145222 0.014907

TS (290.5 kJ/mol)
-1647.904471
h 1.809506 -1.374718 0.406761
o 0.975395 -0.691483 -0.127367
co -0.823274 -0.094751 -0.026309
o -2.540689 0.518106 0.088657
h -3.155037 -0.212687 -0.028833
c 2.372826 -0.240645 0.171691
o 2.732415 0.872073 -0.048506

TS (106.5 kJ/mol)
-1647.974580
h 0.409845 -1.065034 1.034182
c 1.382350 0.032913 -0.025933
co -0.547802 -0.184089 0.176546
o 1.037527 1.236095 0.052136
o 2.382938 -0.580777 -0.321912
o -2.307657 -0.017460 -0.421261
h -2.815756 0.735087 -0.117015

TS (8.5 kJ/mol)
-1648.011883
co 0.834943 -0.053601 -0.036602
o -0.968865 -0.458122 -0.256466
c -2.158450 -0.278494 0.186775
o -2.905826 0.671243 0.000482
h -2.533116 -1.142722 0.786717
o 2.623772 0.298639 0.025872
h 2.947708 0.166832 0.921773

TS (220.6 kJ/mol)

-1647.931114
Co -0.439052 -0.038883 0.000001
O -2.863488 -0.003554 0.000002
C 1.518846 -0.051332 0.000007
O 1.460435 1.201406 -0.000000
O 2.401195 -0.887805 -0.000005
H -2.621917 -0.561302 0.762734
H -2.621908 -0.561234 -0.762777

TS (50.6 kJ/mol)
-1647.931114
Co -0.439052 -0.038883 0.000001
O -2.863488 -0.003554 0.000002
C 1.518846 -0.051332 0.000007
O 1.460435 1.201406 -0.000000
O 2.401195 -0.887805 -0.000005
H -2.621917 -0.561302 0.762734
H -2.621908 -0.561234 -0.762777

TS (305.3 kJ/mol)
-1647.898848
c 0.909428 -0.162388 -0.015986
o 1.565313 -1.099783 0.372871
o 1.538801 0.932225 -0.569408
o -0.504892 0.626523 1.216949
co -1.004123 -0.099122 -0.222133
h -1.623136 -0.749344 -1.573388
h 2.484108 0.728245 -0.496406

TS (143.0 kJ/mol)
-1647.960665
o -1.190744 -1.350397 -0.076449
co 0.574645 0.096438 -0.036623
c -1.266656 0.412210 -0.080750
o -2.359110 0.827550 0.070072
h -1.749955 -1.564681 0.679706
o 2.418429 0.075365 0.187011

IIa
-1724.480605
C -2.631079 0.279253 0.359893
O -1.613262 0.093436 -0.409303
Co 0.170387 -0.369736 -0.090907
O 1.937829 -0.737765 0.257749
O -3.760904 0.579354 0.005491
H -2.421620 0.143989 1.443834
H 2.218039 -1.537791 -0.195639
H 3.263741 0.367462 0.178996
O 4.002406 1.017656 0.062212
H 3.597306 1.712231 -0.461246

IIb
-1724.468659
o -3.097448 0.000288 0.377060
c -1.956775 0.000089 0.142826
co -0.218378 -0.000152 -0.168045
o 3.069739 0.000388 0.464786
h 2.474917 0.751497 0.293576
h 2.475414 -0.751151 0.293858
o 0.763394 -1.619324 -0.236311
o 0.763314 1.619085 -0.236551
h 0.347290 -2.424228 0.070541
h 0.347235 2.423958 0.070417

IIc
-1724.454267
C -1.500245 -0.010661 -0.001408
O -2.761686 -0.593233 0.003957
O -0.558035 -0.874032 0.003409
Co 1.379077 -0.790318 -0.001014
O -1.455547 1.210022 -0.009746
H 2.941831 -0.787488 -0.003951
H -2.596028 -1.542098 0.010054
H 1.617958 1.970164 0.005704
O 0.984909 2.697971 0.005432
H 0.125505 2.236150 -0.000390

IId
-1724.443410
C 1.909196 0.096724 0.121444
Co -0.015756 -0.322867 -0.104157
O -1.824496 -0.736452 -0.280262
O 2.321547 1.352799 -0.342973
O 2.787932 -0.607674 0.594340
H -2.019443 -1.125043 -1.137590
H 3.281211 1.368587 -0.173987
H -3.202178 0.162774 0.176301
O -3.972827 0.753808 0.388444
H -3.586593 1.630907 0.342469

IIe
-1724.442092
C 1.913245 0.303881 -0.053406
O 2.784227 -0.668947 -0.608033
O 2.448234 1.348355 0.243396
Co 0.005347 -0.198034 0.173510
O -1.789662 -0.665026 0.349066
H -1.983801 -1.054548 1.206024
H 2.221329 -1.428922 -0.796160
H -3.232553 0.055794 -0.217728
O -4.050180 0.546570 -0.495583
H -3.769768 1.463693 -0.467250

IIf
-1724.444072
C 1.585280 0.501115 0.016342
O 0.554013 1.175878 0.283702
Co 0.136543 -0.795427 -0.034527
O -1.746631 -1.050803 0.027723
O 2.769143 0.691556 -0.103160
H 1.141755 -1.941453 -0.310565
H -2.094162 -1.394381 0.853366
H -2.419898 0.619869 -0.123969
O -2.591274 1.588551 -0.154613
H -1.708047 1.944369 -0.013869

IIg
-1724.417346
C 2.545132 0.007720 -0.075429
Co 0.772154 -0.011866 0.010035
O -0.896851 -0.047676 0.364567
O 3.708808 0.015319 -0.086080
O -2.988650 -1.544123 -0.200547
O -2.946033 1.656061 -0.086308
H -2.131419 -1.033701 -0.039605
H -3.444616 -1.441515 0.637947
H -3.452310 0.944503 -0.490349
H -2.108795 1.168125 0.140577

IIh
-1724.408471
C -0.252833 0.035301 0.020039
O 0.236542 -1.089483 -0.144317
O 0.202536 1.166588 0.171631
Co -2.258563 -0.006724 -0.007143
H 2.134065 1.522911 -0.007726

O 3.101273 1.496115 -0.110974
H 3.262906 0.554510 -0.263085
H 1.993894 -1.373933 -0.110908
O 2.963411 -1.497379 0.020299
H 3.077217 -1.340471 0.961232

IIi
-1724.480312
C 2.760328 -0.437273 0.100349
O 3.129125 0.589455 0.650991
O 1.583581 -0.777103 -0.291408
Co -0.082902 0.099114 -0.221992
O -1.788149 0.797694 -0.250992
O -4.059780 -0.515271 0.417432
H 3.511321 -1.230825 -0.118993
H -1.805935 1.646396 0.200942
H -3.250516 -0.019778 0.126389
H -3.696700 -1.206444 0.975156

IIj
-1724.471539
C 3.159848 0.023380 -0.223016
O 2.598278 1.138759 -0.079610
O 2.662261 -1.120210 -0.093492
Co -1.494655 -0.007963 0.052186
O -3.260632 -0.081207 -0.355063
O 0.355892 -0.024441 0.524344
H 4.239993 0.058180 -0.501204
H 0.984825 -0.769029 0.328493
H 1.003712 0.712906 0.323339
H -3.678323 0.769473 -0.190989

IIk
-1724.458886
C -2.078821 -0.919371 0.019944
O -0.877009 -1.209613 -0.117221
Co 0.937761 0.173846 -0.014380
O -0.499544 1.642564 -0.146711
O -2.598149 0.229326 0.120708
O 2.532926 -0.747766 0.074230
H -2.797051 -1.770965 0.060092
H 3.251417 -0.116329 0.176365
H -1.399847 1.164278 -0.017319
H -0.366926 2.229311 0.601412

IIIa
-1800.942480
C 3.252520 -0.046492 0.389143
O 2.053468 0.023175 0.854404
Co 0.392670 0.101625 -0.021617
O -1.327034 0.167162 -0.718748
O 3.629676 -0.083036 -0.771620
O -3.210956 -1.634982 0.065678
H 4.008359 -0.074182 1.205937
H -1.297696 0.275001 -1.674089
H -2.489814 -1.051759 -0.282597
H -2.839241 -1.993103 0.875007
O -3.727429 1.365278 0.291288
H -4.020281 0.451607 0.393675
H -2.820334 1.226751 -0.037157

IIIb
-1800.935422
C 2.399027 -0.046562 0.181585
Co 0.659640 0.009896 -0.150483
O -0.309368 -1.632652 -0.166831
O 3.538025 -0.069544 0.409627
O -0.197959 1.674864 -0.406172
O -2.889487 1.562740 0.393592
H -2.950725 0.615880 0.579030
H -1.957243 1.680331 0.111030
H 0.290494 2.477466 -0.225775
H 0.143962 -2.439878 0.073348
O -2.959906 -1.485573 0.152235
H -1.971836 -1.593522 0.079260
H -3.209546 -1.126786 -0.702984

IIIc
-1800.914138
C 0.301005 -1.400948 -0.203586
O 1.237996 -0.716757 -0.605933
O 0.550769 -2.746984 -0.018832
O -0.895199 -1.063679 0.073405
Co -1.844511 0.629117 0.175273
O 1.166808 2.094164 -0.721381
H -2.631447 1.973775 0.272148
H -0.279224 -3.117166 0.301038
H 0.298650 2.317331 -0.362885
H 1.125351 1.121001 -0.810155
H 2.800442 1.558370 0.475067
O 3.403628 0.870016 0.791653
H 2.969970 0.072139 0.462645

IIId
-1800.904307
C 2.721367 0.071219 0.113545
O 3.173816 1.307007 -0.362519
O 3.571885 -0.656037 0.601638
Co 0.786688 -0.290560 -0.117353
O -1.039912 -0.651790 -0.298276
O -3.102025 0.671266 0.575179
O -5.686744 0.051278 -0.185689
H -1.255163 -1.041808 -1.149632
H 4.132512 1.297921 -0.187819
H -2.309948 0.131556 0.251405
H -2.833359 1.583474 0.449762
H -5.883402 -0.622002 0.468952
H -4.755570 0.294858 0.011936

IIIe
-1800.902597
C 2.746955 0.055014 0.129258
Co 0.810034 -0.289403 -0.129131
O -1.023635 -0.586231 -0.347066
O 3.258218 1.302513 -0.305850
O 3.570817 -0.690757 0.607871
O -3.108556 0.594258 0.674019
O -5.671797 0.055806 -0.222206
H -1.247578 -0.921290 -1.219316
H 2.495604 1.779400 -0.653097
H -2.308215 0.109549 0.292336
H -2.856039 1.519441 0.657890
H -5.888950 -0.682933 0.350206
H -4.747853 0.274921 0.028811

IIIf
-1800.904892
C 1.951935 0.966091 0.044757
O 2.988646 1.575974 0.025588
O 0.717207 1.213806 0.097104
Co 1.107561 -0.788125 0.027728
O -0.580380 -1.679377 -0.052799
O -2.202594 0.394190 -0.542668
H 2.488630 -1.489178 -0.002411
H -0.863371 -2.115705 0.753295
H -1.692475 -0.446820 -0.383282

H -1.541953 1.072594 -0.365840
O -4.790699 0.663692 0.383443
H -3.838112 0.577446 0.158084
H -5.225913 0.538217 -0.462374

IIIg
-1800.885530
C 2.930791 -0.000543 -0.001870
O 4.091407 -0.000006 -0.009872
Co 1.153390 0.000168 0.006431
O -0.566665 0.000485 0.000862
O -2.609690 -0.494090 1.730413
O -2.604603 -1.255057 -1.297359
H -1.752047 -0.423393 1.236955
H -3.039219 0.319228 1.440482
H -3.035122 -1.409634 -0.448331
H -1.747842 -0.862064 -0.988876
H -1.749681 1.285606 -0.253865
O -2.606426 1.748488 -0.442338
H -3.034554 1.090432 -1.002449

IIIh
-1800.869847
C 0.885074 -0.519404 0.008382
O 0.996485 0.767941 0.050316
Co -1.058735 -0.909644 -0.064067
O -2.795528 0.293369 0.090719
O 1.822128 -1.322873 0.002071
O 3.937610 0.617833 0.049514
H -2.243356 1.136733 0.035925
H -3.108430 0.247494 1.001337
H 3.127285 1.147857 0.072122
H 3.513679 -0.259515 0.027244
H -0.217974 1.667777 0.040322
O -1.060884 2.275424 -0.019030
H -0.994284 2.682914 -0.886148

IVa
-1877.405902
C -2.692958 -0.790139 0.009119
O -2.372200 0.450061 -0.021609
Co -0.595743 1.110632 -0.012667
O 1.190861 1.571419 -0.001016
O -1.961725 -1.775616 0.054885
O 2.675041 -0.280611 1.461630
O 0.893916 -2.265550 0.057308
H -3.791131 -0.948317 -0.009086
H 1.360149 2.516259 -0.004997
H -0.058070 -2.057272 0.058810
H 1.282630 -1.713547 0.752903
H 3.127371 -0.516581 0.640224
H 2.124857 0.471360 1.157046
H 1.981917 -1.177470 -1.247491
O 2.584060 -0.469036 -1.532242
H 2.135454 0.333997 -1.211773

IVb
-1877.400617
C -2.710388 -0.236288 -0.013370
Co -0.965393 0.074236 0.032418
O -0.292764 1.837061 -0.020506
O -3.854785 -0.421865 -0.050918
O 0.259095 -1.403811 0.086404
O 2.640631 -0.652370 1.550568
O 2.584327 -1.113853 -1.420532
O 2.429645 1.685641 -0.207010
H 2.645715 0.249713 1.184868
H 1.782853 -0.999555 1.242457
H -0.103059 -2.288686 0.131110
H -0.856504 2.592722 -0.177987
H 1.677927 -1.266377 -1.073853
H 3.095380 -1.156397 -0.601277
H 2.495442 0.975249 -0.861685
H 1.460997 1.860260 -0.142748

IVc
-1877.374367
C -0.416829 -1.449499 -0.035230
O -1.577567 -0.955073 0.089546
Co -2.272292 0.868772 0.082675
O 0.648862 -0.883301 -0.290268
O -0.360594 -2.813029 0.138662
O 1.007736 1.884253 -0.613041
O 3.671625 1.738195 0.586183
H -2.851766 2.316434 0.089210
H -1.263610 -3.085176 0.336297
H 0.178218 2.286821 -0.330526
H 0.832804 0.925349 -0.541083
H 2.786994 1.956075 0.249892
H 3.666839 0.771355 0.612846
H 3.604250 -0.830192 -0.954022
O 3.417130 -1.201321 -0.087859
H 2.441585 -1.258299 -0.069244

IVd
-1877.368391
C 2.750486 -0.101670 0.323530
O 3.365931 1.136533 0.510688
O 3.420196 -1.085220 0.590002
Co 0.895943 -0.049725 -0.377296
O -0.883860 -0.041611 -1.011241
O -2.545936 -1.963054 -0.012015
O -4.442552 0.078137 0.973471
H -0.913445 -0.060356 -1.972526
H 4.261364 0.921998 0.829689
H -1.934760 -1.294275 -0.410568
H -2.040059 -2.336050 0.713669
H -3.901472 0.817779 0.655321
H -3.931117 -0.696362 0.690956
H -1.943883 2.370798 0.611638
O -2.464524 1.993666 -0.101339
H -1.884053 1.281458 -0.468894

IVe
-1877.366220
C -2.784220 -0.116660 -0.334683
Co -0.923705 -0.078192 0.355180
O 0.862190 -0.035983 0.972527
O -3.486714 1.102532 -0.442780
O -3.408120 -1.098450 -0.664681
O 2.575664 -1.947203 0.028509
O 2.425483 2.003141 0.030658
O 4.508699 0.097882 -0.862373
H 0.903357 -0.048821 1.933619
H -2.868611 1.783293 -0.153498
H 1.942958 -1.283723 0.399852
H 2.101324 -2.335877 -0.710062
H 3.943757 0.833328 -0.578325
H 3.988713 -0.680477 -0.607370
H 1.968369 2.273911 -0.769005
H 1.847866 1.294165 0.408148

IVf
-1877.368396
C 2.353290 0.630295 -0.326652
Co 1.103427 -0.815069 0.060945

O -0.671441 -1.152260 0.745457
O 3.437943 0.913646 -0.755509
O 1.346947 1.208327 0.161784
O -1.489760 1.381816 1.203536
O -3.517238 1.510370 -0.669926
H 2.109752 -1.858222 -0.480314
H -0.721493 -1.718282 1.518313
H -1.258901 0.420023 1.126078
H -0.664326 1.809528 0.948240
H -2.814635 1.547048 0.018196
H -3.031421 1.396750 -1.490816
H -3.453958 -0.632827 -0.626819
O -3.086955 -1.519578 -0.739311
H -2.233252 -1.477500 -0.266735

IVg
-1877.348814
C -3.122088 0.000008 -0.000022
Co -1.341583 0.000012 -0.000060
O 0.391983 0.000015 -0.000085
O -4.279691 -0.000003 0.000087
O 2.184337 -2.122911 -0.262942
O 2.184314 -0.262987 2.122535
O 2.184395 2.122877 0.263070
H 1.412584 -0.115503 1.527762
H 2.626341 -0.981993 1.652794
H 2.626765 1.652478 0.981442
H 1.412561 1.528296 0.115510
H 1.412502 -1.528301 -0.115501
H 2.626819 -1.652535 -0.981261
H 1.412742 0.115518 -1.527679
O 2.184479 0.262955 -2.122470
H 2.626420 0.982106 -1.652877

IVh
-1877.333651
Co 0.956052 0.834099 -0.074935
O -2.023274 3.067966 0.027990
H -2.078231 2.085240 -0.000295
H -1.076412 3.211518 0.113052
C -0.766411 -0.145358 -0.000233
O -0.471392 -1.398072 0.058967
O -1.926528 0.302407 -0.017169
O -3.213206 -2.353950 0.039045
O 1.971661 -2.170049 0.018124
H 0.980841 -1.873745 0.064601
H 2.045414 -2.627035 -0.823210
H -2.244761 -2.420837 0.069119
H -3.278594 -1.387787 0.008641
O 2.964479 0.259653 0.072104
H 3.298683 0.421933 0.960797
H 2.724192 -0.721450 0.039460

Va
-1953.867538
C 2.954937 1.130279 -0.008905
O 2.079833 1.991292 0.033010
O 2.829534 -0.144663 -0.037801
Co 1.171470 -1.062591 -0.026339
O -0.545922 -1.751844 -0.010631
O -1.970042 -0.112863 1.781645
O -0.809778 1.965368 0.068079
O -1.977538 0.025992 -1.650934
H 4.015177 1.456400 -0.025218
H -0.598447 -2.709791 0.018231
H 0.165374 1.954882 0.068208
H -1.079322 1.375480 0.794666
H -2.819098 -0.038788 1.318924
H -1.475397 -0.782833 1.265063
H -1.510603 0.790147 -1.257007
H -1.519810 -0.731087 -1.230002
H -3.483399 0.214622 -0.749426
O -4.214841 0.404220 -0.111902
H -4.183758 1.359242 -0.010591

Vb
-1953.865890
C -2.931451 -0.000445 0.000631
Co -1.154769 -0.000049 -0.001250
O -0.203506 1.656610 0.051669
O -4.089285 -0.000574 0.001847
O -0.202176 -1.655919 -0.055900
O 2.211537 1.424409 -1.530825
O 2.234418 -1.472681 -1.402753
O 2.208254 -1.424114 1.534723
H 2.256119 -0.453951 1.621721
H 1.321010 -1.582055 1.162708
H -0.698259 -2.474380 -0.056002
H -0.699941 2.474851 0.052406
H 1.322766 1.582464 -1.162458
H 2.259343 0.454401 -1.619250
H 2.650273 -1.517350 -0.527129
H 1.295650 -1.605854 -1.154040
O 2.230850 1.472686 1.404707
H 1.292424 1.605868 1.154986
H 2.647343 1.516677 0.529290

Vc
-1953.835829
C -1.100682 1.480449 -0.004451
O -1.338199 2.832924 -0.025563
O -2.148821 0.764144 -0.009345
Co -2.414628 -1.168676 0.008883
O 0.081127 1.128621 0.016985
O 3.454365 -0.732501 1.503554
O 0.971744 -1.460827 0.021820
O 3.418585 -0.969758 -1.439176
H -2.612050 -2.715578 0.023020
H -2.297085 2.927399 -0.039969
H 0.194669 -2.035005 0.029972
H 0.593417 -0.552301 0.027415
H 2.508765 -1.261569 -1.264955
H 3.817494 -1.062725 -0.560376
H 2.596948 -1.142110 1.314281
H 3.323466 0.187671 1.213384
H 3.066954 0.986866 -0.779190
O 2.896190 1.642877 -0.085168
H 1.926528 1.695068 -0.041572

Vd
-1953.827693
C 3.275566 -0.266349 0.256954
Co 1.398756 -0.080576 -0.353329
O -0.411724 0.051726 -0.891515
O 4.035494 0.902283 0.254630
O 3.842989 -1.284088 0.615148
O -2.163239 -1.469728 0.445535
O -1.720478 2.351667 -0.236181
O -3.729674 0.886977 1.222053
H -0.514890 -0.098270 -1.836150
H 4.919694 0.624858 0.555941
H -1.497337 -0.940772 -0.074073
H -1.643962 -2.091672 0.959508
H -3.156584 1.509128 0.739343
H -3.200189 0.074770 1.233632
H -1.108411 2.814327 0.340925

H -1.224673 1.542000 -0.517770
H -4.878678 -0.320439 -0.057728
O -4.978935 -1.161237 -0.529011
H -4.110254 -1.561093 -0.390729

Ve
-1953.825231
C 3.304809 -0.273708 0.257034
O 3.823564 -1.271835 0.700033
Co 1.415902 -0.104435 -0.324399
O -0.402261 0.051143 -0.833140
O 4.156177 0.843123 0.139033
O -2.180191 -1.462655 0.486243
O -1.698394 2.355801 -0.160438
O -3.790863 0.886888 1.179694
O -4.937017 -1.150250 -0.652958
H -0.525062 -0.098661 -1.775620
H 3.608979 1.550703 -0.219928
H -1.501188 -0.937848 -0.019091
H -1.677453 -2.093959 1.005200
H -3.195854 1.507516 0.722652
H -3.261606 0.075592 1.221567
H -1.124357 2.766408 0.490388
H -1.204607 1.546590 -0.446387
H -4.863279 -0.314712 -0.167692
H -4.081907 -1.557360 -0.462258

Vf
-1953.830322
C -2.169074 0.928098 -0.118753
O -0.961757 1.043502 -0.469578
Co -1.408324 -0.838673 0.200584
O 0.274760 -1.772950 -0.020627
O -3.162541 1.593716 -0.065818
O 1.332894 -0.333671 -2.105741
O 3.536620 0.770428 -0.648224
O 2.350908 -0.822219 1.550474
H -2.741704 -1.337288 0.806227
H 0.211172 -2.707357 -0.226346
H 1.002847 -0.936480 -1.396241
H 0.705138 0.395962 -2.065718
H 2.886008 0.387267 -1.266511
H 3.004143 1.395981 -0.130979
H 2.968574 -0.528029 0.861852
H 1.630898 -1.267164 1.051462
H 1.685830 0.980058 1.516714
O 1.503949 1.883533 1.192383
H 0.687617 1.793919 0.683334

Vg
-1953.811582
C 3.560916 0.015066 -0.201222
O 4.710814 0.048176 -0.334080
Co 1.792897 -0.035991 -0.003931
O 0.071497 -0.074450 0.166818
O -1.842739 0.216631 -1.659569
O -1.620350 -1.610594 1.464880
O -2.948994 2.477267 -0.620789
O -1.595225 1.383996 1.848559
H -0.876915 -1.147058 0.967300
H -2.012963 -0.855509 1.923970
H -2.393483 -0.559937 -1.474912
H -1.067079 0.112537 -1.030947
H -2.210364 3.085583 -0.701396
H -2.614769 1.645018 -1.043293
H -0.900691 0.964880 1.286967
H -2.209420 1.755515 1.198734
H -2.450635 -2.907637 -1.158073
O -3.077150 -2.330935 -0.714309
H -2.620207 -2.092777 0.133046

VIa
-2030.328702
C 2.778092 1.764624 -0.077544
O 3.037228 0.512794 -0.030204
Co 1.776700 -0.898539 0.049267
O 0.375231 -2.105493 0.119853
O 1.685539 2.329990 -0.081895
O -1.464357 -1.048880 -1.732162
O -3.779044 -0.852939 -0.093956
O -1.495934 -0.749316 1.708688
O -0.950628 1.362361 -0.072581
H 3.691226 2.391793 -0.118772
H 0.632514 -3.023283 0.232030
H -0.034758 1.709300 -0.066194
H -1.011244 0.703386 0.651371
H -2.338909 -0.909482 1.247666
H -0.853235 -1.348067 1.274475
H -1.240890 -0.141467 -1.469776
H -0.830873 -1.576764 -1.203694
H -3.089741 -1.032022 -0.764566
H -3.885551 0.111825 -0.122786
H -2.607945 2.001775 -0.089577
O -3.579818 2.112620 0.009732
H -3.695787 2.296722 0.945082

VIb
-2030.325021
C 3.108344 -0.693560 -0.132824
O 4.212665 -1.027934 -0.203362
Co 1.407486 -0.182504 -0.037882
O 0.939416 1.467307 0.840464
O -0.011065 -1.191727 -0.827554
O -2.031495 0.640679 -1.271594
O -1.751449 0.979612 1.659869
H -2.057708 0.886195 0.741823
H -0.796123 1.154866 1.557325
H 1.610391 1.915467 1.355723
H 0.177595 -1.887074 -1.457748
H -1.322297 -0.041067 -1.240480
H -1.568361 1.490394 -1.347758
H -3.667238 -0.655324 -0.965375
O -4.102425 -1.430510 -0.587890
H -3.452856 -1.739055 0.066712
H -1.256854 3.349553 -0.047396
O -0.577227 3.129873 -0.691132
H 0.060685 2.582297 -0.168092
O -1.844742 -1.950098 1.226624
H -1.869991 -1.072566 1.641060
H -1.178854 -1.832340 0.520564

VIc
-2030.295798
C 1.787075 1.384635 -0.015804
O 0.560870 1.251964 -0.023416
O 2.264879 2.671456 -0.018354
O 2.688395 0.490187 -0.006351
Co 2.596747 -1.458303 -0.003166
O -0.752466 -1.129030 0.012779
O -2.194145 2.106855 0.038715
O -2.822713 -0.111422 1.809337
O -2.829657 -0.040097 -1.689731
H 2.492468 -3.014560 -0.001163
H 3.225196 2.591775 -0.011582
H -0.085734 -1.829575 0.023057
H -0.219182 -0.298884 -0.001850

H -2.083024 -0.589319 1.398973
H -3.601687 -0.431755 1.323596
H -2.089185 -0.574271 -1.356316
H -2.670898 0.824961 -1.257161
H -2.436512 1.520979 0.779238
H -1.223977 2.075141 0.017872
H -4.718089 -2.006453 -0.165884
O -4.820568 -1.054136 -0.099430
H -4.180778 -0.687881 -0.756877

VId
-2030.287913
C 3.578041 -0.190618 -0.003118
O 4.257411 -1.146309 -0.337371
Co 1.614018 0.016634 -0.172355
O -0.269899 0.146463 -0.303756
O 4.259776 0.893472 0.549006
O -1.773300 -1.997187 0.037794
O -1.783692 1.640172 1.257893
O -3.566096 -0.526626 1.819602
O -4.147441 -0.712082 -1.021789
H -0.600525 0.546531 -1.118813
H 5.197747 0.629601 0.535673
H -1.150875 -1.223025 -0.106183
H -1.222880 -2.781987 0.002998
H -3.031616 0.286751 1.795810
H -2.953558 -1.200811 1.486255
H -1.260217 2.206054 1.828942
H -1.124752 1.083905 0.745454
H -4.268578 -0.525672 -0.074613
H -3.395405 -1.323510 -0.996984
H -3.228752 0.924355 -1.548065
O -2.665469 1.713127 -1.644576
H -2.497642 1.984163 -0.732616

VIe
-2030.285418
C -3.589629 0.215345 0.004385
O -4.362800 -0.899555 0.388715
O -4.217487 1.227166 -0.205897
Co -1.623889 0.001607 -0.160018
O 0.260066 -0.159358 -0.258157
O 1.791544 -1.571086 1.361423
O 1.781088 1.996099 -0.049456
O 3.614515 0.602004 1.750465
O 4.110557 0.627653 -1.110262
O 2.575378 -1.792748 -1.565304
H 0.597755 -0.598865 -1.050572
H -3.734283 -1.619269 0.515278
H 1.151433 1.221010 -0.140529
H 1.235910 2.782797 -0.113285
H 3.070734 -0.204684 1.784577
H 2.999108 1.266117 1.402605
H 1.280961 -2.086682 1.988788
H 1.124500 -1.040327 0.832584
H 4.258598 0.493085 -0.158019
H 3.369167 1.252204 -1.097718
H 3.157162 -1.012085 -1.524103
H 2.448837 -2.030163 -0.637640

VIf
-2030.291763
C 2.280185 0.341969 0.552138
O 3.311283 0.943996 0.638961
O 1.316144 -0.007661 1.289871
Co 1.116772 -0.683258 -0.628295
O -0.442253 -1.823137 -0.681008
O -2.447061 -0.151236 -1.591298
O -1.362269 -1.659318 1.884984
O -0.561212 1.830786 -1.769601
O -3.074638 0.532209 1.115453
H 1.974715 -0.245148 -1.842891
H -0.302239 -2.720505 -0.988994
H -1.076319 -1.818166 0.952923
H -0.601113 -1.208424 2.265053
H -2.617742 -0.246651 1.483137
H -2.396949 1.231851 1.157475
H -2.833249 0.093614 -0.726892
H -1.817139 -0.868448 -1.363033
H -1.260968 1.134280 -1.822874
H 0.253109 1.412370 -2.072720
H -0.660952 2.255813 0.044896
O -0.788569 2.293484 1.013685
H -0.106504 1.702598 1.356651

VIg
-2030.275584
C 3.797864 -0.008090 0.033708
Co 2.017390 -0.016413 0.062266
O 0.282158 -0.022034 0.035756
O 4.954587 -0.002509 0.003193
O -1.309147 -1.989923 0.855686
O -1.353590 0.286385 -2.035285
O -2.626511 -2.314402 -1.472746
O -2.524594 2.560793 -1.189141
O -1.312134 1.678127 1.350117
H -0.646777 -1.289517 0.574882
H -1.871199 -1.532045 1.502191
H -1.898712 -0.514640 -1.973176
H -0.673475 0.170397 -1.305327
H -1.911907 3.222057 -1.519064
H -2.167236 1.703321 -1.544529
H -0.652281 1.072605 0.901467
H -1.781587 2.091926 0.607602
H -2.044829 -2.900593 -1.962224
H -2.191966 -2.232020 -0.581057
O -3.020902 -0.158736 2.400117
H -2.408870 0.556415 2.085700
H -3.726837 -0.157817 1.748527

VIIa
-2106.788205
C 3.036126 1.788267 -0.273042
O 1.966963 2.238163 -0.681385
O 3.289469 0.629272 0.207844
Co 2.047849 -0.780970 0.425892
O 0.669251 -2.012764 0.543607
O -0.595649 1.123059 -0.918002
O -3.422760 1.586962 -0.848855
O -1.595033 -0.673979 1.269438
O -0.694275 -1.607544 -1.955325
O -3.307643 -1.318996 -0.865900
H 3.928307 2.444288 -0.308082
H 0.914523 -2.856445 0.930537
H 0.293022 1.527754 -0.829733
H -0.762261 0.660588 -0.080430
H -2.221840 -1.030827 0.606403
H -0.771097 -1.205667 1.170453
H -0.561050 -0.646986 -1.889717
H -0.196229 -1.938310 -1.182900
H -2.506555 -1.517073 -1.389930
H -3.471281 -0.368093 -1.006098
H -2.472020 1.666979 -1.037752
H -3.468935 1.661351 0.119009
H -2.553941 0.683338 1.949278
O -3.265425 1.343196 2.088621

H -4.018516 0.816744 2.367791

VIIb
-2106.783567
C 3.419090 -0.053101 -0.067506
O 4.573756 -0.064896 -0.096947
Co 1.641253 -0.044084 -0.002134
O 0.615588 -1.500767 0.681762
O 0.627315 1.504134 -0.551439
O -0.950654 2.372280 1.401045
O -1.833298 -0.428512 1.398630
H -1.635794 0.488495 1.643462
H -0.957780 -0.857652 1.267502
H 1.021854 -2.211525 1.177665
H 1.037885 2.191769 -1.076262
H -0.309799 2.118763 0.676975
H -0.422352 2.785962 2.086505
H -2.408507 3.110665 0.087046
O -2.944398 3.148333 -0.716149
H -2.784336 2.279785 -1.115940
H -2.994853 -2.181629 1.021716
O -3.152239 -3.019151 0.568340
H -2.517468 -2.985389 -0.167032
O -1.879854 0.421450 -1.382066
H -2.082896 0.091598 -0.487300
H -0.994918 0.821733 -1.268831
O -1.054522 -2.470060 -1.422550
H -1.386468 -1.603111 -1.701190
H -0.406484 -2.243066 -0.726678

VIIc
-2106.756703
C 1.860964 1.333594 -0.008899
O 2.790532 0.473615 -0.001589
Co 2.801267 -1.478135 -0.009611
O 0.635259 1.157336 -0.012309
O 2.285545 2.636456 -0.014842
O -0.524650 -1.338224 0.024606
O -1.912552 2.012141 -1.380496
O -2.631807 -0.673185 -1.824503
O -4.636755 -1.288041 -0.085119
O -2.637479 -0.721157 1.906365
H 2.759782 -3.035795 -0.017358
H 3.248669 2.599938 -0.010642
H 0.202160 -1.978121 0.017870
H -0.069838 -0.465101 0.013835
H -1.886845 -1.084024 1.406623
H -3.408656 -0.906489 1.340606
H -1.876178 -1.050740 -1.341724
H -2.463153 0.291445 -1.785399
H -2.158805 2.101089 -0.438084
H -0.952974 1.911608 -1.316658
H -4.751832 -2.235799 -0.184797
H -3.978156 -1.039393 -0.779848
H -0.960407 1.854608 1.268431
O -1.903666 1.974025 1.443697
H -2.219167 1.081118 1.693543

VIId
-2106.747885
C 3.771912 -0.173615 -0.094465
O 4.452505 0.048342 1.102324
O 4.447993 -0.526481 -1.046094
Co 1.816339 0.145449 -0.052790
O -0.062187 0.395742 -0.067648
O -1.573321 -0.157936 1.990999
O -1.428320 -1.261761 -1.590668
O -2.716299 -2.478561 0.744212
O -4.184860 -0.681670 -0.949229
O -3.560890 1.518319 0.748990
H -0.351806 1.293477 -0.298311
H 5.386813 -0.133875 0.893792
H -0.872331 -0.617777 -1.051991
H -0.841932 -1.598309 -2.271473
H -2.355886 -1.807205 1.349876
H -2.157211 -2.376580 -0.042372
H -1.073938 -0.067961 2.805322
H -0.924602 0.052765 1.244305
H -4.009928 -1.360504 -0.272805
H -3.364932 -0.725055 -1.466943
H -3.897081 0.824802 0.147579
H -2.967528 1.032869 1.347668
H -2.360766 2.443281 -0.219079
O -1.629695 2.836744 -0.747446
H -1.840884 2.612737 -1.656989

VIIe
-2106.745465
C 3.786649 -0.211179 -0.090224
Co 1.830744 0.118719 -0.071672
O -0.047527 0.375266 -0.072333
O 4.562165 0.229058 1.002164
O 4.407427 -0.756142 -0.973592
O -1.588723 -0.046322 1.996293
O -1.424542 -1.362338 -1.505043
O -2.761950 -2.420333 0.877752
O -4.177832 -0.696296 -0.936609
O -3.542733 1.583880 0.642797
O -1.564730 2.795912 -0.877058
H -0.335888 1.257452 -0.359938
H 3.939242 0.638226 1.613361
H -0.865111 -0.694106 -1.003235
H -0.839892 -1.744228 -2.163094
H -2.396858 -1.719865 1.446042
H -2.193134 -2.374774 0.092950
H -1.101925 0.086137 2.812270
H -0.927670 0.114032 1.248085
H -4.028130 -1.340072 -0.221233
H -3.351332 -0.785004 -1.437844
H -3.881209 0.863797 0.074522
H -2.963889 1.122672 1.273806
H -2.315286 2.441173 -0.348224
H -1.781350 2.574747 -1.785950

VIIf
-2106.752131
C 2.104673 -0.153945 0.978767
Co 1.302656 -0.467278 -0.739416
O -0.319846 -1.031306 -1.716346
O 3.072013 0.303997 1.533749
O 0.943441 -0.534098 1.331799
O -1.209696 -2.604050 0.342206
O -2.463861 0.536166 -1.382262
O -1.256105 2.550826 -0.316714
O -1.300923 1.064665 2.224084
O -3.228937 -0.723266 1.012538
H 2.714198 -1.018167 -1.071600
H -0.159964 -1.367158 -2.599686
H -0.938860 -2.162736 -0.499104
H -0.527533 -2.309758 0.957803
H -2.640684 -1.484978 0.837965
H -2.677652 -0.130389 1.555931
H -2.885758 0.092379 -0.615627
H -1.692327 -0.054291 -1.609192
H -1.778855 1.800130 -0.765949
H -1.656478 3.375207 -0.599542

H -1.294182 1.709052 1.500411
H -0.495735 0.538048 2.077719
H 0.388799 2.124134 -0.770693
O 1.256648 1.733355 -1.019180
H 1.903386 2.058397 -0.385805

VIIg
-2106.734947
C 3.846092 -0.649477 0.157173
O 4.960993 -0.877049 0.356173
Co 2.125886 -0.275133 -0.124468
O 0.454936 0.164606 -0.339081
O -1.534961 0.578304 1.251744
O -0.794650 3.409977 1.273013
O -0.520493 2.467182 -1.282216
O -3.031180 0.591011 -1.251256
O -1.093372 -1.337838 -1.987958
O -2.385586 -1.712024 2.386180
H -0.065108 1.616136 -1.012182
H -1.399574 2.168599 -1.551369
H -1.426996 1.504667 1.518356
H -0.708563 0.375691 0.687018
H -1.572885 -2.220476 2.441866
H -2.090140 -0.818310 2.085362
H -0.465555 -0.851321 -1.389253
H -1.610763 -1.918506 -1.411884
H 0.109233 3.374835 1.595367
H -0.715984 3.170020 0.315607
H -2.393284 0.005096 -1.707033
H -2.655277 0.666658 -0.355265
O -3.396737 -2.304288 -0.266731
H -3.138347 -2.171938 0.662814
H -3.613821 -1.414748 -0.580764

VIIIa
-2183.248420
C -3.506293 1.661578 -0.046097
O -3.720381 0.405099 -0.165340
Co -2.387978 -0.938849 -0.174535
O -0.913477 -2.063381 -0.158452
O -2.436434 2.253336 0.087134
O 1.003320 -0.643356 -1.516896
O 3.410437 1.216671 -1.947441
O 0.214798 1.389945 0.410229
O 3.267177 -0.491553 1.759480
O 0.535873 -0.963459 2.039728
O 2.910098 2.147560 0.795441
H -4.437355 2.261631 -0.072403
H -1.109842 -2.979469 -0.368141
H -0.705745 1.697473 0.274158
H 0.419979 0.786574 -0.327741
H 1.837817 -1.045408 -1.232127
H 0.298941 -1.225799 -1.148057
H 0.321378 -0.073543 1.707068
H 0.080532 -1.536366 1.392461
H 2.335418 -0.755235 1.929192
H 3.216654 0.467777 1.563592
H 1.936878 2.106630 0.754446
H 3.179430 2.004061 -0.127977
H 2.487141 1.016231 -2.146853
H 3.750459 0.360657 -1.637056
H 3.612967 -1.069918 0.270979
O 3.734638 -1.314301 -0.694536
H 4.240104 -2.128328 -0.717286

VIIIb
-2183.243152
C -3.461702 -1.111711 -0.368757
O -4.501731 -1.578832 -0.549729
Co -1.848449 -0.405436 -0.101969
O -1.717850 1.371598 0.667227
O -0.213612 -1.275539 -0.563257
O 1.635850 0.504763 -1.576368
O -0.323113 2.831715 -1.323826
H 0.143818 2.112620 -1.773755
H -0.886949 2.372578 -0.667129
H -2.531622 1.809403 0.918631
H -0.219463 -2.107342 -1.037612
H 1.003093 -0.160038 -1.215511
H 1.870130 1.091198 -0.832236
H 3.211329 -0.390821 -1.784686
O 4.080964 -0.845476 -1.762265
H 4.642773 -0.249039 -1.260587
H 1.256724 2.973867 -0.162513
O 2.006811 2.615236 0.344460
H 1.594242 2.279610 1.157054
O 3.909564 -2.288232 0.888465
H 3.902114 -1.973755 -0.026446
H 3.072311 -1.965768 1.263256
O 0.346303 1.401906 2.344513
H 0.077818 1.636452 3.234658
H -0.481260 1.418743 1.784156
O 1.292609 -1.396288 1.814696
H 1.122615 -0.490500 2.109446
H 0.774314 -1.466964 0.987644

VIIIc
-2183.215176
C -2.053399 1.121504 -0.565823
O -2.498752 2.271199 -1.152013
O -2.925480 0.220952 -0.412900
Co -2.871805 -1.544627 0.425142
O -0.849514 1.097767 -0.253515
O 0.435745 -1.282046 0.395392
O 3.025450 -0.609085 1.558865
O 4.452658 -1.946213 -0.340800
O 2.943973 2.045185 0.740146
O 1.752366 1.488404 -1.761116
O 2.156353 -1.311735 -1.951126
H -2.759842 -2.944744 1.097483
H -3.436379 2.135453 -1.330385
H -0.270584 -1.902044 0.630222
H -0.041422 -0.449254 0.190491
H 1.509588 -1.485769 -1.247719
H 3.011373 -1.539774 -1.543207
H 2.125937 -0.878203 1.309749
H 3.069036 0.344969 1.322008
H 2.646582 1.915275 -0.184018
H 2.148210 2.368403 1.194163
H 4.410344 -2.867367 -0.074066
H 4.022685 -1.448802 0.398042
H 0.832698 1.500903 -1.461716
H 1.942875 0.543829 -1.933827
H -0.142724 2.136359 1.131111
O 0.318659 2.496914 1.910991
H 0.299073 1.775941 2.546397

VIIId
-2183.212947
C 4.129086 -0.110814 -0.003821
Co 2.149688 -0.051412 -0.094332
O 0.255190 -0.033881 -0.141800
O 4.770740 1.122059 0.106333
O 4.846052 -1.095543 -0.049299
O -1.025909 1.821996 1.205175
O -1.147009 -2.004266 0.886898

O -2.701791 -0.163009 2.472587
O -4.564119 0.032211 0.303216
O -3.008960 2.031318 -0.843948
O -1.679681 0.300198 -2.410586
H -0.121327 0.053245 -1.030155
H 5.721370 0.908004 0.115453
H -0.551759 -1.288444 0.501608
H -0.564132 -2.729012 1.123339
H -2.153274 0.603863 2.236275
H -2.199018 -0.913190 2.112141
H -0.401440 2.457375 1.561675
H -0.473338 1.143613 0.704243
H -4.064331 -0.041864 1.139053
H -4.217272 -0.702256 -0.238854
H -3.667386 1.413964 -0.450941
H -2.356924 2.165183 -0.133679
H -2.174215 0.999225 -1.904633
H -1.568487 0.635835 -3.302090
H -2.458768 -2.120903 -0.527280
O -3.109361 -1.917306 -1.221564
H -2.647011 -1.281852 -1.792361

VIIIe
-2183.210272
C 4.145432 0.121838 0.022897
O 4.820056 1.107766 0.208901
Co 2.165063 0.036027 0.095574
O 0.269981 -0.018067 0.120274
O 4.868848 -1.056353 -0.251696
O -1.140036 2.172524 -0.283018
O -1.043197 -1.391644 -1.693578
O -3.079777 1.481556 1.738692
O -3.001345 -2.188223 0.235169
O -1.640507 -0.984353 2.212844
O -4.564804 0.054595 -0.259051
O -2.734881 0.872126 -2.307454
H -0.107026 -0.352807 0.947919
H 4.205994 -1.745239 -0.374346
H -0.542845 1.377889 -0.128946
H -0.559430 2.936380 -0.303966
H -2.185374 0.070487 -2.309503
H -2.224332 1.487346 -1.754253
H -0.431465 -1.887780 -2.241397
H -0.476791 -0.892358 -1.025618
H -4.079491 0.369208 -1.046005
H -4.207689 0.602423 0.466242
H -3.663685 -1.483841 0.048732
H -2.361842 -2.110223 -0.494180
H -2.147945 -1.507621 1.535987
H -1.532648 -1.555588 2.975675
H -2.439301 1.879920 1.124375
H -2.610128 0.708629 2.092761

VIIIf
-2183.213577
C 1.947819 -0.801289 0.652799
O 0.866050 -0.652817 1.314244
Co 0.697413 -1.178484 -0.737062
O 1.084295 0.758304 -1.622483
O 3.112839 -0.574118 0.900783
O -1.188205 -1.496187 -1.221225
O -2.975853 0.500247 -1.088174
O -1.159267 2.339709 -1.221815
O -1.812025 -1.979152 1.435103
O -2.978544 0.544549 1.758413
O -0.176427 1.996143 1.507681
H 1.732728 -2.304805 -0.946835
H -1.322392 -2.134860 -1.923338
H -1.712535 -1.953440 0.454985
H -0.913579 -1.784875 1.734455
H -2.623853 -0.370829 1.765307
H -2.202620 1.104438 1.901316
H -3.159418 0.520767 -0.128074
H -2.373395 -0.285035 -1.208796
H -1.930248 1.684732 -1.229912
H -1.449012 3.134993 -1.671942
H -0.516433 2.167558 0.613604
H 0.145622 1.075133 1.495349
H 0.281149 1.313317 -1.658086
H 1.722764 1.286868 -1.103026
H 1.820287 2.370529 0.851234
O 2.598307 2.234753 0.287222
H 3.014500 1.430852 0.639637

VIIIg
-2183.196146
C 4.194574 -0.181835 -0.259894
Co 2.423692 -0.058601 -0.081692
O 0.689703 0.111946 0.105556
O 5.341081 -0.250740 -0.375404
O -0.296543 2.667926 0.858404
O -2.624834 2.125480 -0.985158
O -0.833280 0.580446 -2.006790
O -2.425176 -1.796884 -1.946014
O -0.681133 -2.313402 0.127246
O -2.566434 -1.368463 2.131658
O -0.392930 0.308916 2.607090
H -0.208691 0.393710 -1.235470
H -1.317065 -0.255564 -2.150013
H -1.120296 2.698608 0.354685
H 0.139595 1.847937 0.532866
H 0.068480 0.110749 1.750543
H -0.583676 1.249453 2.487845
H -0.146167 -1.481330 0.115684
H -1.289021 -2.190145 0.877969
H -2.937362 2.729037 -1.662385
H -1.905802 1.554006 -1.430713
H -1.806374 -2.112477 -1.246206
H -3.140804 -1.382626 -1.445557
H -1.865572 -0.737464 2.404037
H -3.067238 -0.901182 1.447614
O -4.065741 -0.044600 -0.114073
H -4.959497 0.195513 0.138006
H -3.625353 0.790006 -0.393962

IXa
-2259.709728
C -3.738324 1.725844 0.272193
O -2.698124 2.374182 0.330424
O -3.899721 0.481683 0.006818
Co -2.605133 -0.818403 -0.407935
O -1.240223 -2.028978 -0.767803
O 0.962862 -0.411639 -1.221941
O 3.481237 0.735997 -2.019403
O 2.370856 -1.334544 1.093580
O 0.072875 1.898188 0.213280
O 0.093412 -2.926212 1.408417
O 1.970167 1.164640 2.143375
O 2.910318 2.740009 -0.052308
H -4.696170 2.247206 0.467678
H -1.496072 -2.720101 -1.384240
H -0.893180 2.046462 0.219324
H 0.235453 1.134204 -0.370594
H 1.484514 -0.754986 -0.472032
H 0.179887 -1.002163 -1.254660
H -0.448548 -2.647366 2.150499

H -0.422231 -2.648592 0.606553
H 1.647900 -1.953512 1.320884
H 2.214181 -0.492613 1.582393
H 1.945070 2.788567 -0.123192
H 3.171685 2.122409 -0.763287
H 2.587527 0.357863 -2.047254
H 4.000632 0.072632 -1.531850
H 2.571217 1.691468 1.584418
H 1.112622 1.381202 1.728442
O 4.767540 -1.176269 -0.251131
H 5.299401 -0.652070 0.352727
H 3.945085 -1.365260 0.248805

IXb
-2259.703040
C -3.251002 -1.713269 -0.123331
Co -1.839516 -0.630397 0.020878
O -0.030279 -1.175565 -0.339615
O -4.158220 -2.417133 -0.228801
O -2.189025 1.221862 0.481081
O -0.282611 2.105842 2.080016
O 1.309964 0.912981 -1.674401
O 3.840902 -0.419171 -1.502728
O 1.306661 -0.341640 2.027200
O -1.193421 2.636035 -1.783573
O 1.060334 3.353023 -0.146734
O 4.034221 -0.779339 1.384528
H -0.528628 2.009921 -2.106435
H -1.624035 2.162444 -1.043230
H -3.098356 1.468608 0.653118
H 0.054070 -1.944897 -0.906237
H 0.852209 0.198625 -1.180398
H 1.400156 1.663612 -1.057923
H 3.038769 0.109241 -1.675488
H 4.039970 -0.262972 -0.563455
H 0.257024 3.401397 -0.694562
H 0.729666 3.071946 0.721021
H 3.870854 -1.675151 1.058288
H 3.151774 -0.497583 1.695519
H -0.628214 2.414917 2.919451
H -1.069929 1.807355 1.536550
H 0.869547 0.517466 2.137945
H 0.874923 -0.732109 1.243986
H 1.958024 -2.463867 -0.139807
O 2.785549 -2.828505 -0.473506
H 3.152527 -2.095746 -1.009815

IXc
-2259.675790
C 2.138193 1.511911 -0.029846
O 0.925574 1.418466 -0.271869
O 2.726488 2.711971 -0.300930
O 2.916767 0.627058 0.443441
Co 2.488259 -1.235347 0.823783
O -0.547031 -0.177503 1.436158
O 0.354931 -0.386607 -2.647915
O -2.381830 0.264892 -2.312402
O -2.416152 -1.504956 -0.238173
O -4.570272 -0.688117 1.289307
O -1.852198 2.525979 -0.658125
O -2.827646 1.550793 1.873660
H 1.958126 -2.682814 1.099778
H 3.657828 2.611649 -0.072892
H 0.095959 -0.819471 1.770454
H -0.021960 0.377024 0.815817
H -2.040591 1.000108 2.001621
H -3.535292 0.903948 1.702836
H -1.778107 -1.053979 0.347668
H -2.478189 -0.908976 -1.028348
H -2.295580 1.108818 -1.826284
H -1.474054 0.069922 -2.617106
H -4.600621 -1.291149 2.035637
H -3.892112 -1.068480 0.685082
H -0.896768 2.419102 -0.549258
H -2.237040 2.297272 0.209340
H 0.628883 0.204106 -1.932096
H 0.339436 -1.275119 -2.242251
H -0.844249 -2.609249 -0.872328
O 0.016370 -2.860806 -1.248636
H 0.642166 -2.849189 -0.506879

IXd
-2259.669047
C 4.458905 -0.405959 0.042120
O 5.318050 0.380334 0.401081
Co 2.493601 -0.158547 0.110699
O 0.619543 0.140254 0.154298
O 4.900867 -1.628664 -0.458428
O -0.290395 2.587802 -0.149962
O -0.898014 -0.872088 -1.720198
O -2.397102 2.224774 1.767845
O -3.040250 -1.282874 0.041302
O -1.526699 -0.516428 2.181651
O -4.031739 1.223104 -0.338931
O -2.022685 1.741521 -2.284601
H 0.188298 -0.157569 0.968216
H 5.872634 -1.586524 -0.399677
H 0.123139 1.677796 -0.025319
H 0.438331 3.211891 -0.124378
H -1.654710 0.843252 -2.324598
H -1.411623 2.205576 -1.686521
H -0.405714 -1.505420 -2.246987
H -0.252965 -0.523081 -1.025142
H -3.467844 1.479445 -1.095823
H -3.608041 1.660757 0.426075
H -3.496560 -0.415511 -0.108436
H -2.346002 -1.313353 -0.646207
H -2.097620 -0.888176 1.466728
H -1.576012 -1.133126 2.914957
H -1.658732 2.506413 1.199400
H -2.110979 1.363064 2.111414
H -4.260826 -2.702730 0.088613
O -4.961376 -3.381275 0.100590
H -5.717049 -2.937876 -0.291095

IXe
-2259.666279
C -4.471620 0.401174 0.024431
O -4.991219 1.669072 -0.299385
O -5.297456 -0.452397 0.250045
Co -2.505546 0.152217 0.099177
O -0.628013 -0.132526 0.129254
O 0.914862 0.812700 -1.759285
O 0.284057 -2.596312 -0.105427
O 3.035581 1.277676 0.017355
O 4.036181 -1.233595 -0.281098
O 1.495458 0.566835 2.156754
O 4.908515 3.419205 0.048333
O 2.052926 -1.812165 -2.236703
O 2.373465 -2.183218 1.827062
H -0.193909 0.176483 0.937878
H -4.225721 2.235149 -0.448894
H -0.130268 -1.685056 -0.009979
H -0.444316 -3.220523 -0.071803
H 1.683866 -0.916038 -2.303895
H 1.434142 -2.262222 -1.636182

H 0.432397 1.422178 -2.321810
H 0.258504 0.489209 -1.062463
H 3.484233 -1.513394 -1.038391
H 3.603579 -1.652492 0.489386
H 3.496901 0.408057 -0.102730
H 2.350968 1.287169 -0.679961
H 2.077153 0.920447 1.441239
H 1.538667 1.198390 2.877736
H 1.641945 -2.481576 1.258702
H 2.081863 -1.313473 2.144671
H 4.229349 2.719248 0.043135
H 5.685256 2.989337 -0.316246

IXf
-2259.676651
C -1.399837 1.011645 0.957258
O -2.595593 0.944316 1.191410
O -0.390701 0.460281 1.503602
Co -0.103366 1.542816 -0.327180
O 1.797686 1.582687 -0.808106
O -0.912379 0.287912 -1.844444
O 2.899481 -0.795146 -1.441237
O 0.650242 -2.011459 -1.853627
O 2.566171 1.068503 1.793576
O 3.082870 -1.643148 1.254474
O -3.504277 -0.397119 -1.160377
O 0.083055 -2.272330 0.976059
H -0.877497 2.831403 0.016109
H 2.082502 2.323353 -1.345701
H 2.446318 1.370386 0.863128
H 1.654508 1.003854 2.106237
H 2.977684 -0.717596 1.561006
H 2.193412 -2.021074 1.323250
H 3.131480 -1.132165 -0.552087
H 2.558895 0.125304 -1.275698
H 1.567200 -1.590204 -1.766461
H 0.706552 -2.682389 -2.536482
H 0.223099 -2.282621 0.013457
H -0.040118 -1.330413 1.203219
H -0.397488 -0.531202 -1.980791
H -1.844797 0.011883 -1.698132
H -3.372460 -1.177739 -0.591234
H -3.497280 0.319456 -0.504380
H -1.935960 -2.339759 1.094480
O -2.868869 -2.065611 1.097943
H -2.847619 -1.177467 1.486203

IXg
-2259.652113
C -4.697716 -0.250652 -0.140493
O -5.852723 -0.286594 -0.174039
Co -2.918111 -0.193487 -0.069518
O -1.183004 -0.108242 0.049734
O 0.127489 0.578311 2.222299
O 1.923932 -1.836346 1.849100
O 0.490119 -2.076778 -0.257709
O 1.904514 -0.930124 -2.469599
O 0.144927 1.131804 -1.981791
O 2.300324 2.662858 -0.731232
O 1.041564 3.056489 1.774756
O 4.136084 0.342927 -0.934957
H -0.217636 -1.351204 -0.161278
H 1.004722 -1.812143 -1.044923
H 0.817117 -0.098178 2.251057
H -0.418434 0.338178 1.405548
H 0.246512 3.509578 1.483171
H 0.731876 2.131659 1.977502
H -0.364125 0.737545 -1.224007

H 0.776952 1.753360 -1.581030
H 1.637311 -2.512734 2.466399
H 1.347133 -1.954358 1.007010
H 1.245603 -0.195549 -2.420699
H 2.710973 -0.532426 -2.114317
H 1.951735 2.803751 0.170698
H 2.886969 1.895353 -0.670667
H 5.009893 0.672676 -1.154310
H 4.267757 -0.356394 -0.256051
H 3.530991 -1.805538 1.270839
O 4.399563 -1.781591 0.795429
H 4.347632 -2.517206 0.179074

Xa
-2336.172826
C -3.562561 -2.144756 -0.443087
O -3.948592 -0.946520 -0.198360
Co -2.946252 0.549865 0.336726
O -1.851344 1.974704 0.824229
O -2.430116 -2.615196 -0.401277
O -0.561936 3.265115 -1.165739
O 0.552452 0.712584 1.315484
O 1.813804 1.810281 -0.991023
O 1.644988 -0.548688 -2.281696
O 4.180922 1.327942 0.206973
O 0.209699 -1.719258 -0.130906
O 2.790552 -2.552492 0.883384
O 2.998172 -0.155555 2.480423
H -4.400214 -2.812387 -0.725176
H -2.255740 2.558081 1.471760
H -0.716173 -2.029459 -0.197753
H 0.192383 -0.928598 0.440611
H 1.025311 1.120092 0.564754
H -0.317456 1.168691 1.325646
H -1.036320 3.022638 -1.964837
H -1.075104 2.835424 -0.431378
H 1.056174 2.410697 -1.144680
H 1.693684 1.010145 -1.565527
H 1.871444 -2.482901 0.571716
H 2.876936 -1.838180 1.541892
H 2.089355 0.158872 2.333923
H 3.523454 0.373208 1.860840
H 2.540922 -0.879670 -2.067703
H 1.057502 -1.035423 -1.670602
H 4.812173 2.048381 0.158328
H 3.358300 1.650943 -0.228745
H 3.767108 -1.843560 -0.498509
O 4.163198 -1.305985 -1.218244
H 4.366049 -0.460276 -0.793623

Xb
-2336.159393
C -2.492788 -2.498596 -0.866804
O -3.045730 -3.421965 -1.276620
Co -1.637632 -1.060523 -0.233837
O -2.648925 0.176783 0.870085
O 0.222472 -0.750464 -0.691526
O 3.090455 0.049065 -0.909761
O 2.639194 2.479326 -2.359254
O -0.964084 1.132998 2.671491
O 0.097457 1.949889 -1.455808
O 3.852808 -0.538223 1.883498
O 1.022851 -0.960788 2.105867
O -2.799381 2.556929 -0.615226
O -0.515898 3.365463 0.906247
H -2.114152 2.385825 -1.275470
H -2.850002 1.715003 -0.113742
H -3.521820 -0.113263 1.137899

H 0.551366 -1.391802 -1.325483
H 0.144526 0.991886 -1.243046
H 0.045618 2.411105 -0.593181
H 1.688987 2.368347 -2.123847
H 2.945566 3.197201 -1.799470
H -1.413094 3.325879 0.527652
H -0.543047 2.715176 1.628291
H 3.765516 -0.091746 1.028263
H 2.925984 -0.714676 2.125947
H -1.343433 1.188999 3.550801
H -1.674767 0.756869 2.075256
H 0.464268 -0.230167 2.413349
H 0.833270 -1.016188 1.152305
H 2.155100 -0.149653 -0.739840
H 3.079057 0.854313 -1.457996
H 4.553868 -2.065486 0.614860
O 4.700054 -2.390316 -0.286280
H 4.269783 -1.701500 -0.809821

Xc
-2336.135892
C -2.079255 1.632844 0.531529
O -3.044284 0.977738 0.030536
Co -2.993753 -0.792574 -0.774799
O -0.875217 1.332373 0.555285
O -2.424155 2.815045 1.114021
O 0.116864 -0.177859 -1.524567
O 2.024992 -2.030764 -0.544488
O 2.442440 -1.395548 2.057698
O 4.060523 -0.421096 -1.340737
O -0.331898 -1.031687 2.368171
O 1.766402 2.687051 0.280563
O -0.685911 -3.177349 0.481629
O 2.214102 1.592978 -2.348330
H -2.742014 -2.205205 -1.406574
H -3.380519 2.892906 1.020459
H -0.659251 -0.645183 -1.867511
H -0.238555 0.343480 -0.769198
H 1.432997 1.017403 -2.314457
H 2.948591 0.993440 -2.139966
H 1.345314 -1.393994 -0.837150
H 2.163613 -1.855295 0.423278
H 2.887786 -0.530908 1.950642
H 1.525840 -1.203429 2.336854
H 4.793355 -0.891125 -1.743368
H 3.385458 -1.106737 -1.101481
H 0.870145 2.389935 0.498298
H 1.890610 2.453519 -0.658367
H -0.521547 -0.240946 1.843394
H -0.561542 -1.781779 1.787735
H 0.156761 -3.072145 0.016826
H -1.373497 -2.900880 -0.147943
H 4.063617 0.595000 0.448323
O 3.766002 0.969494 1.289659
H 3.080835 1.621364 1.035095

Xd
-2336.127850
C 4.616195 -0.680605 0.030512
O 5.068185 -1.388369 -1.081445
O 5.455341 -0.369159 0.857643
Co 2.669323 -0.315140 0.090048
O 0.819538 0.111534 0.154835
O -0.323423 0.681994 -2.124756
O 0.155929 2.383696 1.306116
O -2.828813 -0.227497 -1.330054
O -3.525301 2.116612 -0.170545
O -1.752118 -1.210234 1.112583
O -4.458559 -2.389890 -1.330325
O -1.111178 3.289627 -1.104067
O -2.342280 1.334712 2.280330
H 0.262029 -0.558028 0.576077
H 6.026037 -1.494748 -0.937369
H 0.463972 1.514597 0.901695
H 0.910499 2.726343 1.790365
H -0.805017 2.536971 -1.636549
H -0.617631 3.187375 -0.271381
H 0.254716 0.420752 -2.844956
H 0.162618 0.427983 -1.274142
H -2.796009 2.663167 -0.525930
H -3.239772 1.913099 0.744235
H -3.167699 0.628817 -0.961175
H -2.000327 -0.001471 -1.796168
H -2.092157 -0.910118 0.247191
H -2.259054 -2.018637 1.294943
H -1.486131 1.772632 2.134204
H -2.167673 0.403596 2.051043
H -3.865768 -1.609516 -1.434065
H -5.335437 -2.072688 -1.556497
O -3.841192 -3.233445 1.267293
H -4.426503 -2.719824 1.829746
H -4.118624 -3.014551 0.353507

Xe
-2336.125141
C -4.620848 -0.661557 -0.022051
Co -2.672913 -0.291347 -0.085016
O -0.822624 0.140206 -0.115488
O -5.100505 -1.643592 0.866043
O -5.465791 -0.118901 -0.695258
O 0.361637 0.704115 2.142255
O -0.156471 2.421764 -1.265672
O 2.846257 -0.236327 1.309248
O 3.553131 2.098946 0.143750
O 4.408400 -2.450017 1.282147
O 3.692030 -3.302871 -1.285726
O 2.315918 1.346829 -2.290233
O 1.173018 3.303804 1.120375
O 1.704152 -1.189972 -1.113255
H -0.260884 -0.516324 -0.552771
H -4.323357 -1.986770 1.321156
H -0.467529 1.555736 -0.860617
H -0.914253 2.775927 -1.736563
H 0.865014 2.552771 1.653923
H 0.662165 3.214214 0.296876
H -0.205500 0.458952 2.876458
H -0.144048 0.452982 1.301788
H 2.838604 2.655981 0.512618
H 3.249834 1.904094 -0.767100
H 3.191847 0.615861 0.936120
H 2.028216 0.000883 1.787566
H 2.072699 -0.897772 -0.256778
H 2.178262 -2.016895 -1.301981
H 1.468355 1.795512 -2.128916
H 2.132474 0.417287 -2.062395
H 3.845704 -1.649003 1.393995
H 5.300961 -2.162540 1.485570
H 4.298226 -2.844655 -1.873283
H 4.003735 -3.082400 -0.383404

Xf
-2336.138392
C -1.176630 -0.805238 -1.100404
Co -0.134600 -1.383109 0.383260
O -0.742643 0.170668 1.672747
O -2.303030 -0.627645 -1.545160

O -0.033085 -0.472787 -1.536590
O 1.690537 -1.708633 1.014773
O 3.224903 0.459934 1.557189
O 1.234734 2.120952 1.691387
O 2.771798 -1.595031 -1.523416
O -3.071194 1.472123 1.162548
O 3.697853 1.037199 -1.175508
O 0.798726 2.221051 -1.172085
O -2.196808 2.361147 -1.421586
H -1.113976 -2.568438 0.254647
H 1.798990 -2.441970 1.622376
H 2.506860 -1.769724 -0.589966
H 1.923209 -1.418787 -1.948187
H 3.450088 0.118888 -1.413808
H 2.899303 1.557688 -1.344426
H 3.562519 0.672567 0.663835
H 2.712974 -0.382424 1.434367
H 2.057357 1.535403 1.717915
H 1.351225 2.816476 2.341116
H 0.909076 2.294155 -0.208399
H 0.565254 1.288575 -1.330674
H -0.069563 0.863696 1.815578
H -1.596124 0.644638 1.513573
H -2.858093 1.891096 0.306518
H -3.622174 0.698089 0.941550
H -1.237082 2.510945 -1.394149
H -2.288089 1.459953 -1.766684
H -3.586240 -1.001484 -0.370700
O -4.189021 -1.055121 0.403022
H -3.733694 -1.664821 0.991366

Xg
-2336.113068
C 5.023183 -0.013835 -0.373664
Co 3.256769 -0.018785 -0.133329
O 1.522659 -0.060760 0.055220
O 6.166393 -0.023197 -0.540005
O 0.156889 -1.484725 1.758356
O -1.274758 -2.813384 0.124811
O -0.119591 -0.840084 -1.794154
O -1.944336 0.883394 -2.489377
O -3.805088 -1.945483 0.296299
O 0.424895 2.125138 1.231727
O -3.872910 0.886418 1.035553
O -1.224580 0.742278 2.921244
O -1.948881 2.746371 -0.301261
H 0.751534 -0.939841 1.139114
H -0.365049 -0.821129 2.248752
H -0.529777 -1.594431 -1.348377
H 0.554617 -0.490485 -1.110852
H -1.729051 1.135027 -3.389702
H -1.232655 0.220601 -2.211140
H 0.848385 1.359981 0.756916
H -0.263995 2.453478 0.628676
H -0.973831 -3.723662 0.145124
H -0.709825 -2.300431 0.817241
H -0.601719 1.307799 2.399251
H -2.086210 0.920944 2.528620
H -1.889833 2.214507 -1.113344
H -2.521836 2.215404 0.272777
H -4.312221 0.977860 0.172452
H -3.667607 -0.061861 1.030769
H -2.897097 -2.332452 0.232924
H -4.290795 -2.502660 0.907586
H -4.429480 -0.701873 -1.226758
O -4.582273 0.143037 -1.672674
H -3.712767 0.381397 -2.044086
